# A semi‐supervised Support Vector Machine model for predicting the language outcomes following cochlear implantation based on pre‐implant brain fMRI imaging

**DOI:** 10.1002/brb3.391

**Published:** 2015-10-12

**Authors:** Lirong Tan, Scott K. Holland, Aniruddha K. Deshpande, Ye Chen, Daniel I. Choo, Long J. Lu

**Affiliations:** ^1^ Division of Biomedical InformaticsCincinnati Children's Hospital Research Foundation3333 Burnet AvenueCincinnatiOhio45229; ^2^ Department of Electrical Engineering and Computing SystemUniversity of Cincinnati812 Rhodes HallCincinnatiOhio45221‐0030; ^3^ Pediatric Neuroimaging Research ConsortiumCincinnati Children's Hospital Medical CenterCincinnatiOhio45221; ^4^ Department of Speech‐Language‐Hearing‐Sciences, 106A Davison Hall110 Hofstra University, HempsteadNew York11549; ^5^ Department of OtolaryngologyCollege of MedicineUniversity of CincinnatiMedical Sciences Building231 Albert Sabin WayCincinnatiOhio45267; ^6^ Department of Environmental HealthCollege of MedicineUniversity of Cincinnati231 Albert Sabin WayCincinnatiOhio45267

**Keywords:** Cochlear implantation, language outcomes, machine learning, pre‐implant fMRI, semi‐supervised SVM

## Abstract

**Introduction:**

We developed a machine learning model to predict whether or not a cochlear implant (CI) candidate will develop effective language skills within 2 years after the CI surgery by using the pre‐implant brain fMRI data from the candidate.

**Methods:**

The language performance was measured 2 years after the CI surgery by the Clinical Evaluation of Language Fundamentals‐Preschool, Second Edition (CELF‐P2). Based on the CELF‐P2 scores, the CI recipients were designated as either effective or ineffective CI users. For feature extraction from the fMRI data, we constructed contrast maps using the general linear model, and then utilized the Bag‐of‐Words (BoW) approach that we previously published to convert the contrast maps into feature vectors. We trained both supervised models and semi‐supervised models to classify CI users as effective or ineffective.

**Results:**

Compared with the conventional feature extraction approach, which used each single voxel as a feature, our BoW approach gave rise to much better performance for the classification of effective versus ineffective CI users. The semi‐supervised model with the feature set extracted by the BoW approach from the contrast of speech versus silence achieved a leave‐one‐out cross‐validation AUC as high as 0.97. Recursive feature elimination unexpectedly revealed that two features were sufficient to provide highly accurate classification of effective versus ineffective CI users based on our current dataset.

**Conclusion:**

We have validated the hypothesis that pre‐implant cortical activation patterns revealed by fMRI during infancy correlate with language performance 2 years after cochlear implantation. The two brain regions highlighted by our classifier are potential biomarkers for the prediction of CI outcomes. Our study also demonstrated the superiority of the semi‐supervised model over the supervised model. It is always worthwhile to try a semi‐supervised model when unlabeled data are available.

## Introduction

Approximately 1–6 infants per 1000 are born with severe to profound sensorineural hearing loss (SNHL) (Northern 1994; Bachmann and Arvedson 1998; Kemper and Downs 2000; Cunningham and Cox 2003). If left untreated, hearing loss can have detrimental effects on the speech, language, and communication abilities of children (Ching et al. 2009; Luckner and Cooke 2010; Pimperton and Kennedy 2012; Yoshinaga‐Itano 2014). These children will have difficulties in developing language abilities due to their inability to detect acoustic‐phonetic signals. Cochlear implantation, a surgical procedure that implants an electronic device (the cochlear implant or CI) into the cochlea, is effective for restoring hearing, even in severely to profoundly deaf patients with hearing thresholds of 75 dB HL and above (Geers et al. 2009; Hayes et al. 2009; Moog and Geers 2010; Geers and Hayes 2011). In infants and toddlers with prelingual or congenital SNHL, more than two decades of accumulated data show that many of these children can develop and continue to maintain good speech and language abilities with the use of a CI, even in the long term (Beadle et al. 2005; Geers and Sedey 2011; Geers et al. 2011; Ruffin et al. 2013). However, variability in speech and language outcomes among this age group of CI patients remains high and individual outcomes may be difficult to predict (Zaidman‐Zait and Most 2005; Lazard et al. 2010; Niparko et al. 2010; Tobey et al. 2013). According to the Food and Drug Administration, approximately 38,000 children in the United States received a CI as of December 2012 (NIDCD, 2013). While many congenitally deaf CI recipients achieve near‐normal language skills, about 30% of the recipients do not derive the expected benefit from the surgery (Niparko et al. 2010). The reasons underlying the varied benefits across different individuals are not always clear. Furthermore, current behavioral methods used to predict language outcomes for a CI candidate prior to surgery may be inaccurate, particularly in infants. Improved prognostic information would be helpful to clinicians and parents in setting expectations during the CI decision process, particularly given the high medical cost and anesthetic risks of this surgery. The motivation for this study using fMRI and machine learning classification of pre‐implant brain activation to auditory stimulation in CI candidates is to develop a neurobiological biomarker for speech and language outcomes.

Numerous studies investigating factors that influence language outcomes following cochlear implantation have been reported in the literature. Nikolopoulos et al. (1999) first studied the influence of age at implantation with 126 prelingually deafened children younger than 7 years of age at the time of implantation. Regression analysis and Spearman rank correlation coefficients revealed that language outcome was negatively correlated with age at implantation. Since then, several studies investigating the influence of age at implantation on speech and language outcomes have been published (Baumgartner et al. 2002; Manrique et al. 2004; Svirsky et al. 2004, 2007; Connor et al. 2006), for different age of participants, etiology of deafness, and method for measuring language skills. In addition, mutations in gap junction protein beta2 (GJB2) were found to be a common cause of SNHL. Influence of GJB2 mutations on cochlear implantation outcomes was analyzed in (Bauer et al. 2003; Cullen et al. 2004; Sinnathuray et al. 2004a,b), by comparing the language performances between groups with and without GJB2 mutations. Other influencing factors include inner ear malformation (Eisenman et al. 2001; Kim et al. 2006), meningitis (El‐Kashlan et al. 2003), communication mode (oral vs. total) (Osberger et al. 1998; Osberger and Fisher 2000; Kirk et al. 2002), pre‐implant speech recognition skills (Zwolan et al. 1997; Osberger and Fisher 2000), pre‐implant residual hearing (Gordon et al. 2001; Niparko et al. 2010), parent–child interactions (Niparko et al. 2010), and socioeconomic status (Niparko et al. 2010). Approximately 50% of the variability in post‐implant speech perception outcomes was explained by factors like duration of hearing loss before implantation, length of implant use, mode of communication, and implant characteristics (Sarant et al. 2001). Although a variety of influencing factors have been investigated, a predictive model combining these variables has not been developed. Furthermore, despite extensive pre‐implant social, behavioral and clinical work‐ups by pediatric cochlear implant teams, there continues to be variability in outcomes that does not appear to be accounted for by any of these parameters.

Within the past decade, functional Magnetic Resonance Imaging (fMRI) has been discussed as a way to assess auditory function in the brains of children as well as adults (Scheffler et al. 1998; Anderson et al. 2001; Lazeyras et al. 2002; Patel et al. 2007; Propst et al. 2010). With improvements in acquisition, preprocessing, and analysis, it has been suggested that pre‐implant fMRI could be translated into an objective predictor for CI outcomes (Patel et al. 2007). Indeed, the hypothesis motivating the design for our original fMRI study in infants with congenital SNHL was that the pre‐implant cortical activation patterns revealed by fMRI during infancy would correlate with auditory performance 2 years after the CI surgery. Meanwhile, machine learning methods have begun to demonstrate success for analyzing neuroimaging data and show promise for translation of neuroimaging findings in populations to making predictions for individual patients (De Martino et al. 2008; Pereira et al. 2009; Cuingnet et al. 2011). In this work, we attempted to develop a machine learning model based on pre‐implant fMRI data to predict the language outcomes 2 years after cochlear implantation in congenitally deaf infants with SNHL. The Support Vector Machine (SVM) model we developed uses pre‐implant fMRI data from an individual CI candidate to predict whether or not the candidate will develop effective language skills within 2 years after the cochlear implantation. This type of prognostic model could be extremely useful and is currently not available to clinicians by any other means.

## Materials and Methods

### Participants

Forty‐four infants and toddlers participated in a clinically indicated MRI brain study with sedation. This study was conducted under the approval of Cincinnati Children's Hospital Medical Center Institutional Review Board (IRB). Twenty‐three participants had SNHL (12 females, average age = 20.0 months, range = 8–67 months). All the SNHL children received the CI surgery. Their MRI data were acquired before the surgery. The remaining 21 participants were normal hearing (NH) controls (16 females, average age = 12.1 months, range = 8–17 months). They received clinical MRI scans with sedation for indications not likely to be related to the auditory system or temporal‐parietal regions of the brain. Inclusion criteria for the control group included: gestational age of at least 36 weeks, normal otoacoustic emissions, and normal neuroanatomy determined by the neuroradiologist upon review of anatomical MR images. NH children who did not meet these criteria were excluded from this study. As a result, the average age of the NH children was not perfectly matched to the average age of the SNHL children, even though we required the NH subjects to have ages matched to the SNHL group when we were collecting the data. However, this age difference will not invalidate our analysis as discussed in the Discussion section. Informed consent of parent or guardian was obtained prior to the study protocol.

### Cochlear implantation outcomes

Two years after the CI surgery, we administered a battery of tests to assess hearing, speech, language, and cognitive function in the CI recipients. The tests were used to evaluate the CI recipients' auditory, speech, and language outcomes following CI at a point in development when standardized behavioral measures of these skills could be used. For this study, we used the data from the Clinical Evaluation of Language Fundamentals‐Preschool, Second Edition (CELF‐P2) (Wiig et al. 2004) as the primary language outcome measure of interest. The CELF‐P2 detects language delay or language impairment in children between the ages of three and 7 years. The subtests of CELF‐P2 focus on different language domains such as word structure, sentence structure, expressive vocabulary, concepts, and following directions. These subtests help in the assessment of both receptive and expressive components of language. CELF‐P2 is standardized on more than 1500 children including children with hearing impairment (HI). Additionally, age equivalent norms are available for direct comparison between the target and control populations. CELF‐P2 is routinely used in the clinic as a diagnostic as well as therapeutic tool to evaluate and monitor the progress in children's language abilities. Thus, this valid and reliable test (Friberg 2010) was used in this study at the 2‐year follow‐up stage to evaluate CI recipients' language skills post‐implantation. Sixteen of the 23 CI recipients had 2‐year follow‐up scores for the CELF‐P2. Follow‐up scores for the remaining seven children were not available for reasons such as family moving away from the area or a toddler unwilling to comply with the testing during a clinical follow up visit. There were 5 scores/indices for the CELF‐P2, namely the core language score, receptive language index, expressive language index, language content index, and language structure index. The follow‐up scores for the 16 participants are listed in Table 1.

**Table 1:** CELF‐P2 test scores for the CI recipients

Participant index	Core language	Receptive language	Expressive language	Language content	Language structure	Sum	Effective
1	45	45	45	45	45	225	No
2	45	45	45	45	45	225	No
3	45	45	45	45	45	225	No
4	45	45	56	45	59	250	No
5	50	53	50	57	55	265	No
6	53	59	53	53	50	268	No
7	53	59	57	59	57	285	No
8	65	63	65	67	71	331	Yes
9	69	63	75	69	81	357	Yes
10	77	67	69	71	79	363	Yes
11	65	63	77	75	88	368	Yes
12	69	71	75	79	83	377	Yes
13	73	69	81	75	86	384	Yes
14	73	73	83	77	91	397	Yes
15	79	69	81	79	90	398	Yes
16	81	75	87	79	90	412	Yes

CELF‐P2, Clinical Evaluation of Language Fundamentals‐Preschool, Second Edition; CI, cochlear implant.

Each of the five scores/indices of the CELF‐P2 used in this study provides a standard score ranging from 45 to 155 with a mean of 100 and a standard deviation of 15 (Table 1). The score 45 corresponds to a percentile rank of <0.1, whereas a score of 155 corresponds to a percentile rank of >99.9. In children with hearing loss and particularly with severe to profound congenital SNHL, the scores are substantially lower than the maximum value. The five scores/indices are highly correlated with each other. Based on the 16 samples in Table 1, we calculated the pair‐wise correlations between the five scores/indices. The pair‐wise Pearson's correlation coefficient ranged from 0.90 to 0.95, and the Spearman's correlation coefficient ranged from 0.87 to 0.92. Given the continuous outcome scores, a regression model might be more desirable than a classification model. However, regression function estimation is more challenging and requires more samples (Devroye et al. 1996; Wang et al. 2010). Considering our limited sample size as well as the conspicuous gap in the outcome scores (second to the last column in Table 1), we decided to train a classification model at present. To assign the class labels based on the CELF‐P2 scores, we performed a *k*‐means clustering with *k* = 2. The two clusters were labeled as effective and ineffective CI users, respectively. The effective group included nine subjects (4 females, average age = 21.1 months) with high follow‐up scores, whereas the other cluster with seven subjects (3 females, average age = 19.7 months) was ineffective. As shown in Table 1, participants 1–7 were ineffective subjects with class label −1, and the remaining were effective subjects with class label +1. The class labels for the seven children (5 females, average age = 18.7 months) without follow‐up scores were unknown. We then trained classification models to separate the effective from the ineffective CI‐users.

**Figure 1:**
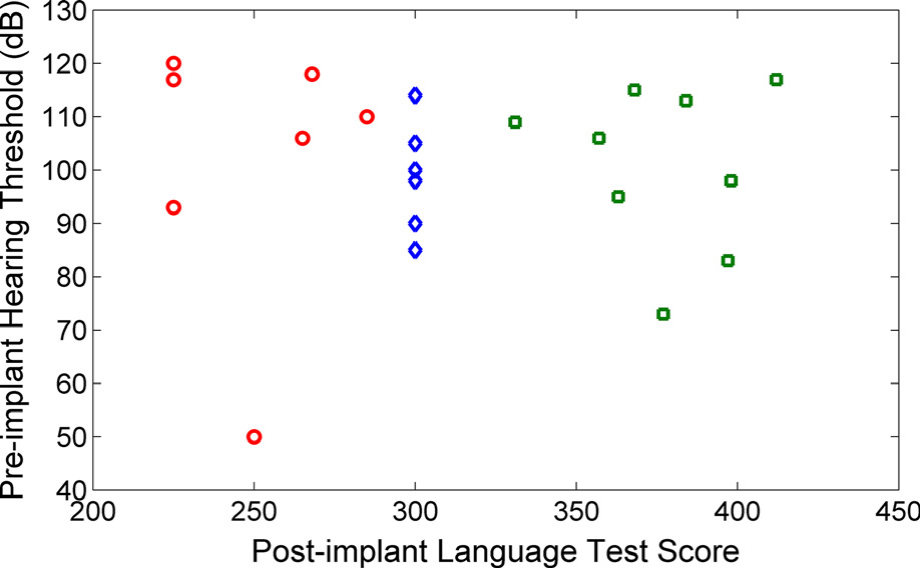
The pre‐implant hearing threshold and post‐implant language test score for the participants. The post‐implant language test score is the sum of the five CELF‐P2 test scores (Table 1). Red circle points represent ineffective CI‐users. Green square points represent effective CI‐users. Blue diamond points represent unlabeled samples. Since the unlabeled samples do not have the post‐implant language test scores, we set their values to be 300 in order to show them in this figure. Furthermore, two of the seven unlabeled samples have the same pre‐implant hearing threshold of 100. They are overlapped in the figure. Thus, it appears that there are only six diamond points in the figure.

It is well‐known that pre‐implant residual hearing is a good indicator for the subsequent success of cochlear implantation. Specifically, children with more residual hearing are likely to be effective CI‐users, whereas those with less residual hearing tend to be ineffective CI‐users. Our present work would be less meaningful, if the effective and ineffective CI‐users in our project could be distinguished merely based on the pre‐implant hearing thresholds. To exclude this possibility, we plotted the pre‐implant hearing thresholds and post‐implant language test scores in Figure 1. Obviously, the effective and ineffective CI‐users were not separable based on the pre‐implant hearing thresholds alone. Furthermore, our previous regression analysis using age at implantation and pre‐implant hearing threshold as independent variables also failed to predict the post‐implant language test scores with a satisfactory accuracy.

### MRI data acquisition & preprocessing

Anatomical images for this study were acquired using a Siemens 3T Trio scanner in the clinical Department of Radiology. Isotropic images of the brain were acquired using an inversion recovery prepared rapid gradient‐echo 3D method (MP‐RAGE) covering the entire brain at a spatial resolution of 1 × 1 × 1 mm in an axial orientation. These high‐resolution 3D‐T1‐weighted images were used for coregistration of fMRI scans which were also acquired during this scheduled MRI.

Functional MRI (fMRI) data were acquired using the silent background auditory stimulation paradigm we have referred to as Hemodynamics Unrelated to Sounds of Hardware (HUSH) paradigm (Schmithorst and Holland 2004). This approach allows auditory stimuli to be presented to participants during silent intervals of the gradients with echo planar images acquired during the peak of the hemodynamic response to the stimulus, 6–12 sec after onset. Three auditory stimuli consisting of noise, speech and silence were presented to the participants via calibrated MR‐compatible headphones. The pre‐implant hearing threshold for each participant was measured using standard audiometry methods to yield a hearing level in decibels for each ear. The sound level of the auditory stimulus was set at an intensity of 10–15 dB above the measured hearing thresholds for each patient using a calibrated, MRI compatible audio system (Avotec Silent Scan Audio System, SS3100; Avotec Inc., Stuart, FL) driven by a computer with discrete setting that was calibrated to the SPL level at the headphones. Auditory stimuli were presented for 5 sec duration and fMRI data were acquired during periods of silence between auditory presentations for 6‐sec (Schmithorst and Holland 2004). A timing diagram for the fMRI data acquisition and stimulation paradigm is appended in the supplementary materials (Fig. S1). Three contrasts can be computed from this three‐phase fMRI data: speech versus silence, noise versus silence, and speech versus noise.

Cortical activation maps for each contrast were created on a voxel‐by‐voxel basis. Data preprocessing was performed using standard procedures in SPM8. Images were realigned to remove residual motion effects (Thevenaz et al. 1998), normalized to the infant template in the MNI space (Friston et al. 1995; Altaye et al. 2008), and smoothed with an 8 mm Gaussian kernel. Finally, the general linear model (GLM) (Worsley et al. 2002) was used to construct three separate contrast t‐maps (speech vs. silence, speech vs. noise and noise vs. silence) for each individual, which were submitted for subsequent machine learning analysis. The reported brain areas and coordinates are in the MNI framework.

### Feature extraction

We used the Bag‐of‐Words (BoW) approach that we previously developed for feature extraction (Tan et al. 2013). The BoW approach was originally developed in document classification for assigning a document to two or more classes. All the words occurring across all of the documents constitute a dictionary. Suppose we have N words in the dictionary. Based on this dictionary, a document can be represented into an N‐dimensional feature vector, with each word becoming a feature. The value of a feature is measured as the occurrence frequency of the corresponding word in the document. We have introduced this idea to the contrast maps. By analogy, each contrast map represents a document, each characteristic contrast region is a word, and all the characteristic contrast regions occurring across all of the contrast maps constitute a dictionary. Given a contrast map, a series of characteristic contrast regions were derived by thresholding the contrast map and subsequently merging the selected voxels into spatially coherent regions. On the basis of the Student's t‐distribution (with the degree of freedom of 60), we had seven thresholds, that is, 1.671, 2.000, 2.390, 2.660, 2.915, 3.232, and 3.460, corresponding to the one‐sided *P*‐values of 0.05, 0.025, 0.01, 0.005, 0.0025, 0.001, and 0.0005, respectively. The seven thresholds gave rise to seven contrast ranges [1.671, 2.000), [2.000, 2.390), ···, [3.460, + ∞). We also considered the corresponding negative contrast ranges, (−∞, −3.460], ···, (−2.390, −2.000], (−2.000, −1.671], because the contrast map speech versus silence included both speech>silence and silence>speech. The positive contrast ranges came from the contrast speech>silence, whereas the negative contrast ranges came from the contrast silence>speech. Each contrast range was considered separately; take the contrast range [1.671, 2.000) for example. We first selected the voxels with contrast value within this range. The selected voxels were then merged into regions by connecting the voxels adjacent to each other in the 3D space, in which each voxel had 26 neighbors if it was not on the border. Voxels that were connected made up a region, that is, a connected component, which was added into the dictionary as a word. We extracted all the words occurring across all the subjects and all the contrast ranges to build a dictionary for each type of contrast map. The dictionary was then applied to each subject to convert his/her corresponding type of contrast map into a feature vector. The value of a feature was calculated as the mean contrast value of the voxels within the corresponding region.

We analyzed two sets of BoW features. BoW21 constructed the dictionaries exclusively from the 21 NH subjects, whereas the BoW44 used all the 44 subjects including the 23 SNHL subjects to construct the dictionaries. Hypothetically, the activated/deactivated brain regions from the NH subjects should be included in the dictionaries, because they represented the actual brain activation pattern in response to external auditory stimuli in sedated infants and were likely to distinguish between effective versus ineffective CI‐users. With those considerations, we did not consider the feature set which constructed the dictionary using the 23 SNHL subjects alone as we expected such a feature set would miss a number of features that would be critical for predicting effective or near‐normal speech and language outcomes. For comparison purposes, we also trained models with voxel features. In this approach, each voxel became a single feature, the value of which was the image intensity of this voxel in the contrast maps (Ryali et al. 2010; Nouretdinov et al. 2011; Rizk‐Jackson et al. 2011; Brodersen et al. 2012; Oliveira et al. 2013; Hart et al. 2014). For convenience, we denoted this approach as VOX.

### Supervised SVM model

The input for the supervised SVM model training is a training set *D* = {(*X*_1_, *y*_1_), ···, (*X*_M_, *y*_M_)}, where M is the number of training samples, *X*_m_ = (*x*_1_, …, *x*_N_) and *y*_m_ ∈ {−1, + 1} are the feature vector and class label for the *m*‐th sample, respectively, N is the number of features. Our learning objective is to estimate the model $\hat{y}=wX+b$, where *w* = (*w*_1_, …, *w*_N_) is the weight vector and *b* is the bias by minimizing the objective function in Equation (1).

(1) $$\begin{array}{cc} & \frac{1}{2}\left\|w^{2}\right\|+C\sum_{i=1}^{M}\xi_{i}\\ & s.t.\forall_{i}^{M}:y_{i}\left({\mathrm{wX}}_{i}+b\right)\geq 1-\xi_{i},\xi_{i}\geq 0 \end{array}$$

where *C* is a regularization parameter controlling the trade‐off between margin and training error, *ξ* is the slack variable.

### Semi‐supervised SVM model

The inputs for the semi‐supervised SVM model training are a set of labeled samples *D*_1_ = {(*X*_1_, *y*_1_), ···, (*X*_M_, *y*_M_)} and a set of unlabeled samples *D*_2_ = {*X*_1_, …, *X*_K_}, where M is the number of labeled samples and K is the number of unlabeled samples. In our project, we had seven unlabeled samples, whose follow‐up scores were missing. The semi‐supervised model has the same format as that of the supervised model, namely $\hat{y}=wX+b$, but with a different objective function as defined in Equation (2).

(2) $$\begin{array}{cc} & \frac{1}{2}\left\|w^{2}\right\|+C\sum_{i=1}^{M}\xi_{i}+U\sum_{j=1}^{K}\xi_{j}^{*}\\ & s.t.\forall_{i}^{M}:y_{i}\left({\mathrm{wX}}_{i}+b\right)\geq 1-\xi_{i},\xi_{i}\geq 0\\ & \forall_{j}^{K}:y_{j}\left({\mathrm{wX}}_{j}+b\right)\geq 1-\xi_{j}^{*},\xi_{j}^{*}\geq 0\\ & \frac{1}{K}\sum_{j=1}^{K}\max\left(0,y_{j}\right)=r \end{array}$$

where the first term measures the margin, the second and third term measure the training errors on labeled samples and unlabeled samples, respectively, *C* is the regularization parameter for the labeled samples, and *U* is the regularization parameter for unlabeled samples, *r* is the ratio of positive samples within the unlabeled samples, which is a user specified parameter.

The training process of a semi‐supervised model includes three steps: (1) Train a supervised model based on the labeled samples, followed by application of this initial model to the unlabeled samples. The K * *r* unlabeled samples with the highest predicted scores are assigned to +1, and the remaining are assigned to −1. (2) Assign a temporary parameter *U*_tmp_ and re‐train a new model with all of the samples, including the unlabeled samples. Switch the labels of a pair of unlabeled samples according to a certain rule to achieve the maximal drop of the objective function in Equation (2). Repeat this process until no pair of the unlabeled samples meets the switching rule. (3) Increase the value of *U*_tmp_ and then repeat step (2). When *U*_tmp_ ≥ *U*, terminate the training process and output the model. In this project, both the supervised and semi‐supervised models were trained using the SVM^light^ package (Joachims 1999). Linear kernel was used for the SVM models.

### Feature selection

The goal of feature selection was to find a subset of features to approximate the relationships between the feature vectors and the response variable, instead of using all the features, which may include irrelevant features. We employed the recursive feature elimination (RFE) approach for feature selection. RFE performed feature selection by removing the irrelevant features iteratively. The algorithm started by training an SVM model with all of the features. Based on the trained model, features were ranked based on their absolute weights. Features with the lowest absolute weights were discarded, and a new SVM model with the new feature set was trained. This process was repeated until the number of features reached the predefined threshold.

### Predicting new subjects

Given a new sample, whose class label was unknown whereas feature vector *X* was available, we calculated the $\hat{y}$ by inserting *X* into the learned model $\hat{y}=wX+b$. If $\hat{y}\geq \delta$, the new sample was predicted to have class label +1. Otherwise, its class label was predicted to be −1. The threshold *δ* was set to be 0 by default. The semi‐supervised model and the supervised model had different *w* and *b*, and consequently their predictions were different.

### Model evaluation

We employed the Leave‐One‐Out Cross‐Validation (LOOCV) approach to evaluate the supervised models as well as the semi‐supervised models when the parameter *r* was already known before the model training. For the supervised model, we performed a LOOCV on the 16 labeled samples. Specifically, we performed 16 rounds of training and testing, each round of which was called onefold of cross‐validation. In the *k*‐th fold, the *k*‐th labeled subject was used for testing, and the remaining 15 subjects were used for training. In this way, each labeled subject was used for testing once. The model was evaluated based on the predictions on the 16 testing samples accumulated across the 16 folds of cross‐validation. For the semi‐supervised model with parameter *r* set to be a predefined value, the evaluation approach was generally the same as that applied to the supervised model. We also performed 16 rounds of training and testing, with each of the labeled samples left‐out for testing once. The only difference was that, for each fold of cross‐validation, the semi‐supervised model had 22 training samples, including 15 labeled samples and seven unlabeled samples, whereas the supervised model had only 15 training samples. Please note the unlabeled samples were used only for training but not for testing, and both the supervised model and semi‐supervised model were tested on the same 16 labeled samples.

**Figure 2:**
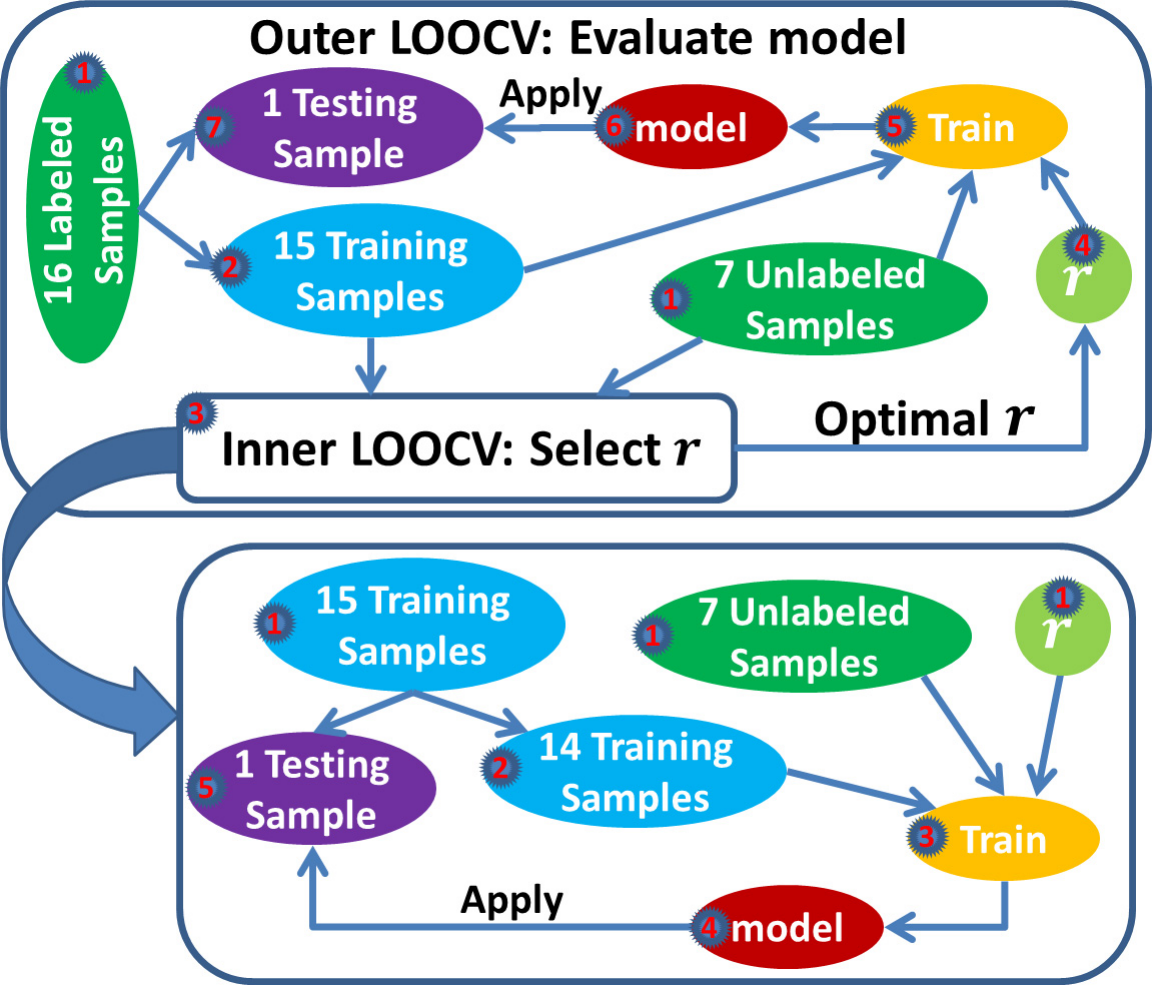
A flowchart for the nested LOOCV that we used to evaluate the semi‐supervised model when the parameter r was unknown before the model training. The outer LOOCV included 16 rounds of training and testing. For each round, one labeled sample was left‐out for testing, and the remaining 15 labeled samples and the seven unlabeled samples were used for training. We submitted these 22 samples to the inner LOOCV box. All the activities in the inner LOOCV box were confined to these 22 samples. The goal of the inner LOOCV box was to optimize the parameter r. Different r values, ranging from 0 to 1 and stepping by 0.1, were tried in the inner LOOCV box. Specifically, we performed a LOOCV on the 22 samples for each value of r. An inner LOOCV included 15 rounds of training and testing. For each round, one labeled sample was left‐out for testing, and the remaining 14 labeled samples and the seven unlabeled samples were used for training. The LOOCV performance was calculated based on the predictions on the 15 testing samples accumulated across the 15 rounds of testing. The r value that achieved the best LOOCV performance was selected. With the optimized parameter r, we jumped out of the inner LOOCV box and returned to the outer LOOCV box. Then we trained a model with the optimized parameter r using the seven unlabeled samples and the 15 labeled samples in the blue ellipse, and applied this model to the left‐out labeled sample for testing. Thus, we completed one round of training and testing for the outer LOOCV. This process was repeated 16 times, with each labeled sample left‐out for testing once. The predictions on those 16 testing samples were used for the evaluation of the model.

For the semi‐supervised model whose parameter *r* was unknown before the model training and needed to be optimized during the model training, we employed a nested LOOCV approach for model evaluation. A flowchart for the nested LOOCV is shown in Figure 2. As we see from Figure 2, the nested LOOCV consisted of an outer LOOCV and an inner LOOCV. The outer LOOCV was used for model evaluation, and the inner LOOCV was used for parameter selection. In the *k*‐th iteration of the outer LOOCV, the *k*‐th labeled subject was used for testing, and the remaining 22 subjects, including 15 labeled samples and seven unlabeled samples, were used for training and parameter selection. Since parameter *r* was the ratio of positive samples within the unlabeled samples, we considered *r* starting from 0 to 1, stepping by 0.1, which gave us 11 different values for *r*. Under each *r* value, we performed an inner LOOCV on the 22 subjects, which was the same as the LOOCV procedure described in the previous paragraph except that we had only 15 instead of 16 rounds of training and testing here, because the 22 subjects only included 15 labeled samples. Then we selected the *r* value that led to the highest F‐measure, which was calculated based on the predictions on the 15 labeled samples. The F‐measure was defined in Equation (3).

(3) $$\begin{array}{cc} & \mathrm{sensitivity}=\frac{\mathrm{true}\mathrm{positive}}{\mathrm{true}\mathrm{positive}+\mathrm{false}\mathrm{negative}}\\ & \mathrm{sensitivity}=\frac{\mathrm{true}\mathrm{negative}}{\mathrm{true}\mathrm{negative}+\mathrm{false}\mathrm{positive}}\\ & F\text{‐measure}=\frac{2*\mathrm{sensitivity}\times \;\mathrm{specificity}}{\mathrm{rm}\mathrm{sensitivity}+\mathrm{specifity}} \end{array}$$

The F‐measure defined as the harmonic mean of sensitivity and specificity promotes a balance between sensitivity and specificity of the classifier, and is often used as a single measure of classification performance. Using the selected *r* value, we trained a model with the 22 subjects, and applied the trained model to the *k*‐th labeled subject for testing. Thus, we completed one iteration of the outer LOOCV. There were 16 iterations for the outer LOOCV, with each of the 16 labeled samples left‐out for testing once. The nested LOOCV was used only when the parameter *r* was tuned automatically. If *r* was predefined to be a fixed value such as 0.6, the inner LOOCV was no longer needed because there was no need to optimize the parameter *r* and the LOOCV as described in the previous paragraph was used for the model evaluation in such a case. More concretely, the nested LOOCV was only used in the section Semi‐supervised model with automatic parameter selection. Based on the results in this section, parameter *r* was set to be predefined values in the remaining analysis of this study, and therefore the LOOCV was used for model evaluation after this section, except for the blind test on NH subjects.

On the basis of the predictions on the testing samples, we calculated the sensitivity, specificity, accuracy, and area under the receiver operating characteristic curve (AUC) to estimate the generalization performance of the models. Furthermore, we calculated the Pearson's correlation coefficient (Pcorr) as well as the Spearman's rank correlation coefficient (Scorr) between the predicted scores and the sum of the CELF‐P2 test scores, which are listed in the second to the last column in Table 1. Pcorr was used to quantify the linear relationship, whereas Scorr was used to measure the monotonic relationship. A model with high correlations was desirable.

## Results

### Supervised model

As defined in Equation (1), the supervised model had one user‐defined parameter, *C*. The effect of the parameter *C* on the classification performance is shown in Fig. S2. As we see, the parameter *C* had negligible effect on the classification performance as long as *C* was larger than a threshold, which was consistent with the previous empirical studies (Abidine and Fergani, 2013; Cherkassky and Ma, 2004). When *C* was smaller than the threshold, the classification performance was generally worse than the stable state. In some cases, for example, BoW21 feature set from the contrast speech versus noise, we may find a parameter *C* that was smaller than the threshold but achieved a slightly better performance than the stable state. For robustness purposes, however, we set *C *= 1 for the supervised models since the classification performance reached the stable state when *C *= 1. The LOOCV performance for the supervised SVM models is shown in Table 2. Among the performance measures, we focused on Pcorr and Scorr. We can see that the contrast speech versus silence outperformed the other two contrasts, which was consistent across the three types of features. The VOX approach obtained almost zero correlations for the contrasts noise versus silence and speech versus noise, which suggested that these two contrasts had very limited predictive power. Combining these two contrasts with the contrast speech versus silence did not improve the classification performance, which implied that including these two contrasts in the training added noisy features and disturbed the classification. In comparison with the VOX approach, our BoW approaches achieved better performance for all the three contrasts. Additionally, the BoW21 returned comparable performance as BoW44, but with fewer features.

**Table 2:** LOOCV performance for the supervised SVM models. The feature vectors from the three contrasts were concatenated to form the feature vector for the “Combine”. Due to the large number of features, we did not train the model with the three contrasts combined for VOX. The best AUC, Pcorr and Scorr across the different feature sets was highlighted in bold

Feature type	Contrast	No. of features	Sensitivity (%)	Specificity (%)	Accuracy (%)	AUC	Pcorr	Scorr
VOX	Speech vs. Silence	26767	33.3	42.9	37.5	0.51	0.29	0.11
	Noise vs. Silence	26767	44.4	42.9	43.8	0.54	0.04	0.04
	Speech vs. Noise	26767	66.7	57.1	62.5	0.59	0.07	0.02
BoW44	Speech vs. Silence	1156	33.3	71.4	50.0	0.56	0.35	0.23
	Noise vs. Silence	1216	55.6	71.4	62.5	0.63	0.21	0.13
	Speech vs. Noise	803	66.7	71.4	68.8	0.67	0.17	0.09
	Combine	3175	77.8	71.4	75.0	0.65	0.15	0.14
BoW21	Speech vs. Silence	658	44.4	85.7	62.5	**0.73**	**0.38**	**0.44**
	Noise vs. Silence	806	55.6	57.1	56.3	0.60	0.21	0.14
	Speech vs. Noise	434	66.7	71.4	68.8	0.68	0.19	0.15
	Combine	1898	66.7	57.1	62.5	0.59	0.16	0.01

LOOCV, Leave‐One‐Out Cross‐Validation; SVM, Support Vector Machine.

### Semi‐supervised model with automatic parameter selection

The semi‐supervised model had three parameters, namely *C*,* U* and *r*, as denoted in Equation (2). The parameter *U* is usually set to be equal to *C*, which is also followed by the SVM^light^ package. Thus, we actually had two parameters, namely *C* and *r*, for the semi‐supervised model. If the two parameters were optimized simultaneously, the computation time would be considerably long, because the number of combinations of the possible values for these two parameters was large. For simplicity, it was acceptable to start with parameter *C* fixed to be a reasonable value (Chapelle et al. 2006; Nie et al. 2013). Based on our previous experience and the results in Figure S2, we started by setting *C *= 1 for the semi‐supervised models. For the experiment in the current section, the parameter *r* was optimized automatically. The model was evaluated using the nested LOOCV approach (Fig. 2). Results are shown in Table 3. In comparison with the supervised models whose performance is shown in Table 2, the semi‐supervised models achieved comparable performance for VOX, and better performance for both BoW21 and BoW44 across all of the three contrasts. We also noticed that the correlations (Pcorr and Scorr) for the contrast speech versus silence were considerably higher than the other two contrasts (Table 3). Furthermore, the correlations for BoW21 were higher than or at least as good as the BoW44 across different contrasts. The BoW21 with the contrast speech versus silence achieved AUC of 0.97, Pcorr of 0.60 and Scorr of 0.76, which significantly surpassed other combinations. Analyzing its automatically selected parameters across different folds of cross‐validation, we noticed that 15 of 16 folds had selected 0.6 for the ratio *r*, which corresponded to four effective and three ineffective children within the unlabeled samples. This ratio was very reasonable, because it was consistent with the ratio on the labeled samples (9 effective vs. 7 ineffective). The labeled samples and unlabeled samples came from the same distribution, and therefore their ratios of positive samples were expected to be close to each other. The only fold that selected a different ratio had selected the ratio 0.7 corresponding to 5 effective and 2 ineffective, which was very close to the ratio selected during other folds. Hence, we were confident that the parameter selection process was very stable across different folds of cross‐validation. For the BoW44 with contrast speech versus silence, the parameter selection was not as stable as BoW21, but it also selected the ratio 0.6 in 12 of 16 folds. The VOX approach for the contrast speech versus silence selected the ratio 0.6 in only four folds. Five other ratios were selected at least once, but none of them were selected for more than four folds. Therefore, the parameter selection was considerably unstable for the VOX approach, which might be a reason why the semi‐supervised model with VOX features did not achieve an improvement in classification performance when compared with the corresponding supervised model. The automatically selected *r* values for the other two contrasts are summarized in Figure S3. As we can see, the most frequently selected *r* values were consistent across different feature sets within the same type of contrast, indicating that our BoW features did not change the general distribution of samples when compared with the VBM features. However, the most frequently selected *r* values varied between different contrasts. This was possible because features from different contrasts were probably very different, and consequently the distributions of samples were different as well. Since the two contrasts noise versus silence and speech versus noise had limited classification performance, it was reasonable to assume that the effective and ineffective CI‐users were not separated as well as the contrast speech versus silence. Consequently, the *r* values relying on the sample distributions from the former two contrasts were likely to be unreliable.

**Table 3:** Nested LOOCV performance for the semi‐supervised models. The parameter ratio *r* was selected automatically

Feature type	Contrast	No. of features	Sensitivity (%)	Specificity (%)	Accuracy (%)	AUC	Pcorr	Scorr
VOX	Speech vs. Silence	26767	44.4	42.9	43.8	0.56	0.23	0.11
	Noise vs. Silence	26767	44.4	85.7	62.5	0.57	0.12	0.04
	Speech vs. Noise	26767	66.7	57.1	62.5	0.63	0.17	0.06
BoW44	Speech vs. Silence	1156	66.7	71.4	68.8	0.73	0.49	0.53
	Noise vs. Silence	1216	55.6	85.7	68.8	0.73	0.37	0.31
	Speech vs. Noise	803	77.8	71.4	75.0	0.71	0.20	0.15
	Combine	3175	77.8	57.1	68.8	0.65	0.19	0.16
BoW21	Speech vs. Silence	658	77.8	85.7	81.3	**0.97**	**0.60**	**0.76**
	Noise vs. Silence	806	55.6	71.4	62.5	0.70	0.37	0.34
	Speech vs. Noise	434	77.8	71.4	75.0	0.75	0.26	0.21
	Combine	1898	77.8	57.1	68.8	0.78	0.32	0.22

LOOCV, Leave‐One‐Out Cross‐Validation.

We also investigated the effect of parameter *C* on the classification performance for the semi‐supervised models. We repeated the above experiments with parameter *C* set to be different values. The results are shown in Figure S4. Since the nested LOOCV was considerably slow, the VBM feature sets were computationally prohibitive for this experiment, and therefore we did not show the results for the VBM feature sets in Figure S4. Consistent with the supervised models, the classification performance had reached the stable state when C=1. Hence, we set C=1 for the remaining analysis in this study.

### Semi‐supervised model with fixed parameter *r*

Since the contrast speech versus silence outperformed the other two contrasts and all the three feature sets selected the ratio *r* of 0.6 most frequently for this contrast, we performed a LOOCV on the contrast speech versus silence with the parameter *r* fixed to be 0.6. Results for this experiment are shown in Table 4. The performance of BoW21 did not change when compared to its performance in Table 3. This was expected because only one fold had changed the ratio from 0.7 to 0.6. However, locking *r *= 0.6 for the BoW44 model changed the ratio in four folds, and achieved better performance. VOX feature set achieved significantly higher AUC and correlations, when comparing its performance in Table 4 with that in Table 3. To make our comparisons more convincing, we used the R package pROC (Robin et al. 2011) to compare two AUCs. A two‐sided *P*‐value was calculated to show if two AUCs were significantly different. We set the significance level at 0.05. In comparison with the supervised models, the AUCs for the semi‐supervised models were significantly higher for BoW44 (*P*‐value = 0.02) and BoW21 (*P*‐value = 0.04), but not for VOX (*P*‐value = 0.09). Comparing among the semi‐supervised models with different feature sets, the AUC for BoW21 was shown to be significantly higher than the AUC of VOX (*P*‐value = 0.03), but not significantly higher than the AUC of BoW44 (*P*‐value = 0.08). Meanwhile, we did not detect significant difference between BoW44 and VOX for the semi‐supervised models. Since the combination of BoW21 and contrast speech versus silence was consistently better than all other combinations, we focused on this feature set in the remaining analysis of this study, with the ratio of positive samples within the unlabeled samples fixed at 0.6 unless stated explicitly as a different value.

**Table 4:** LOOCV performance for the semi‐supervised models with *r* = 0.6 for the contrast speech versus silence

Feature type	Sensitivity (%)	Specificity (%)	Accuracy (%)	AUC	Pcorr	Scorr
VOX	66.7	42.9	56.3	0.70	0.49	0.42
BoW44	77.8	71.4	75.0	0.83	0.55	0.58
BoW21	77.8	85.7	81.3	**0.97**	**0.60**	**0.76**

LOOCV, Leave‐One‐Out Cross‐Validation.

### Why did semi‐supervised model perform better?

**Figure 3:**
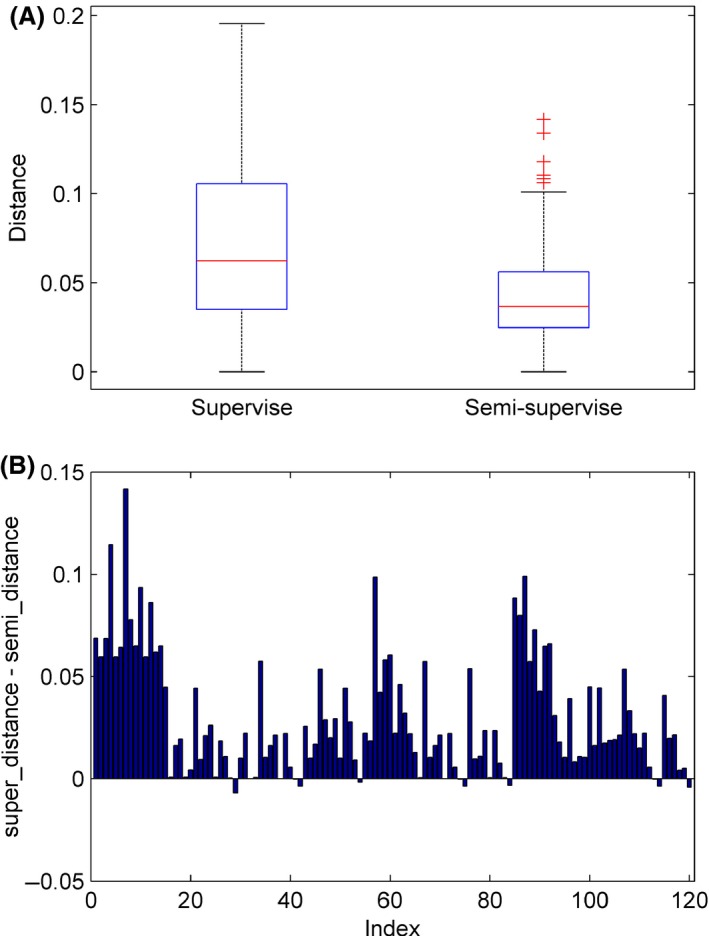
Stability of the weight vectors across different folds of cross‐validation. One‐hundred‐and‐twenty unique pair‐wise distances were calculated among the weight vectors, for the semi‐supervised model and the supervised model, respectively. (A) On each box, the central mark is the median, the edges of the box are the 25th and 75th percentiles, the whiskers extend to the most extreme data points not considered outliers, and outliers are plotted individually. (B) Each bar represents one of the 120 unique distances. *y*‐axis is the distance from the supervised model minus the distance from the semi‐supervised model.

Adding the seven unlabeled samples to the training set increased the sample size by approximately 45%. We speculated that these unlabeled samples contributed to the characterization of the distribution of the two groups of samples, for example, effective and ineffective CI‐users, and consequently helped to identify the accurate hyperplane separating the two groups of samples. To support our speculation, we analyzed the models across different folds of cross‐validation. As described above, the models were defined by the weight vector *w* and the bias *b*. Considering that *w* specified the orientation of the separating hyperplane, we measured the difference/distance between two *w* vectors as 1 − cos *θ*, where *θ* was the included angle between the two *w* vectors. Each fold of cross‐validation yielded a model, and each model had a weight vector *w*. We had 16 models, each of which was trained with one of the 16 labeled samples left out for testing, and then we obtained 120 unique distances by calculating the pair‐wise distance among the 16 weight vectors. Thus, we had 120 distances for the supervised approach and the semi‐supervised approach, respectively. We noticed that the 120 distances were not independent of each other. For example, let three models be *m*_1_, *m*_2_, and *m*_3_. Let the distance between *m*_1_ and *m*_2_ be *d*_12_, the distance between *m*_2_ and *m*_3_ be *d*_23_, and the distance between *m*_1_ and *m*_3_ be *d*_13_. According to the triangle inequality theorem, we would know that $\left|d_{12}-d_{23}\right|<d_{13}<d_{12}+d_{23}$. In other words, *d*_13_ is not independent of *d*_12_ and *d*_23_. Therefore, the paired two sample *t*‐test or Wilcoxon signed‐rank test, which required the samples to be independent to each other, was not appropriate for testing if the means of the 120 distances were significantly different between supervised and semi‐supervised model. A summary of the 120 distances is shown in Figure 3A. As we see, the median and the largest distance were clearly smaller for semi‐supervised model in comparison with supervised model. A detailed pair‐wise comparison is shown in Figure 3B. Among the 120 distances, the semi‐supervised approach was only slightly higher than the supervised approach for 10 distances, suggesting that the weight vectors from the semi‐supervised models were much more similar to each other than that of the supervised models across different folds of cross‐validation. Furthermore, we also calculated the standard deviation (std) for the bias *b* across different folds of cross‐validation. The *std* for the supervised model was 0.08, which was also higher than the 0.04 for the semi‐supervised model. These results confirmed that the semi‐supervised models were more stable and consistent across different folds of cross‐validation.

**Figure 4:**
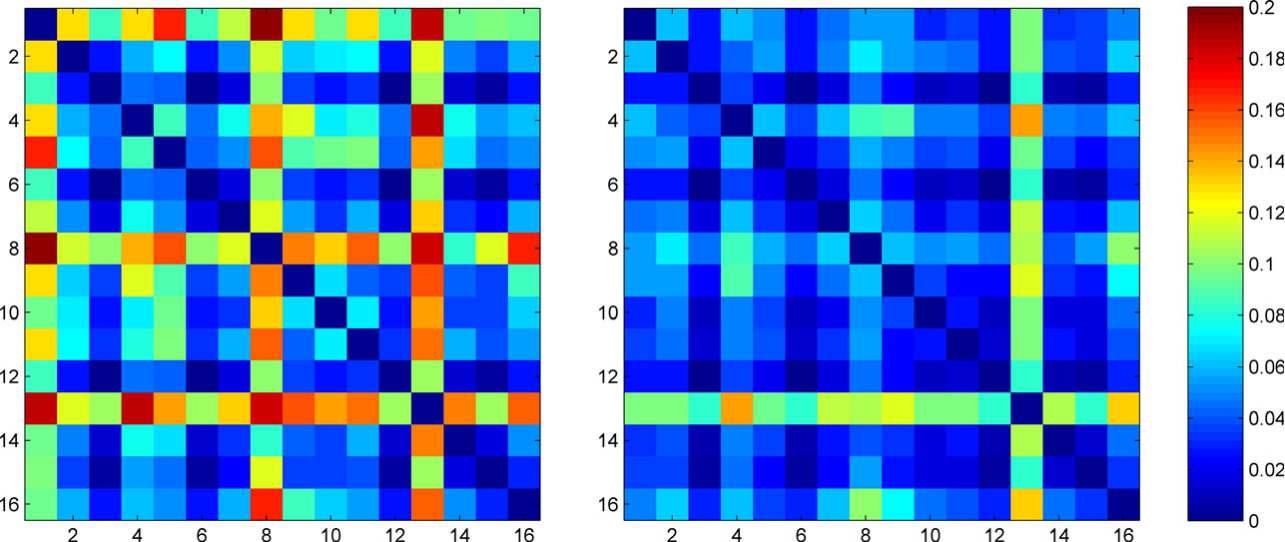
Pair‐wise distance among the weight vectors across different folds of cross‐validation. The left panel is for the supervised model, and the right panel for the semi‐supervised model. Each panel is a symmetrical distance matrix. The color represents the distance, with hot colors representing large distance values and cool colors representing small distance values. The two panels use the same color scale as shown by the color bar.

**Figure 5:**
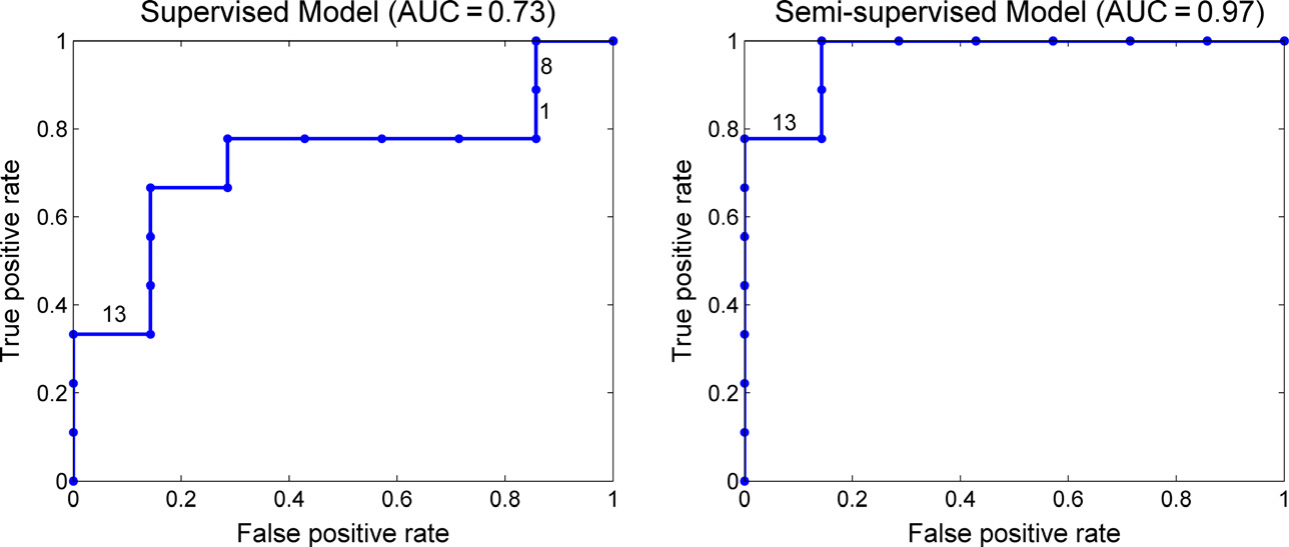
ROC curves for the supervised model and semi‐supervised model. The models were trained on the feature set extracted by BoW21 from the contrast speech versus silence. The parameter r for the semi‐supervised model was fixed to be 0.6. The ROC curves and AUCs were derived based on the predicted values on the testing samples with the LOOCV approach.

Figure 4 shows the distance matrices calculated from the weight vectors as described above. From this figure, we can see that the hyperplane learned by the supervised learning for folds 1, 8, and 13 had considerable deviation from other folds, whereas the semi‐supervised model only had a small deviation for the fold 13. When the hyperplane deviated by a large amount, the predicted value for the testing sample was very inaccurate, which had a great effect on the AUC score, given the limited sample size. AUC, the area under the receiver operating characteristic (ROC) curve, evaluates the ranking of the predicted values for the testing samples. If the predicted value for any negative sample is lower than the predicted value for any positive sample, then it is a perfect ranking and the AUC will reach 1. Figure 5 shows the ROC curves for the supervised model and semi‐supervised model. In the ROC curve, each vertical segment represents a positive sample and each horizontal segment represents a negative sample. As shown in Figure 5, we marked the 1st, 8th, and 13th testing sample on the ROC curve of the supervised model. We can see that the 1st and 8th testing samples were positive samples, but the predicted values for these two samples were lower than almost all negative samples. As a result, the ROC curve cannot climb higher than 0.8 until the false‐positive rate reached as high as 0.8. The 13th sample was a negative sample, but its predicted value was higher than most positive samples. Accordingly, the ROC curve was forced to move horizontally even though the true positive rate was relatively low. Due to the influence of these three samples, the area under the ROC curve cannot be high for the supervised model. Conversely, the ranking of the predicted values from the semi‐supervised model was very close to the perfect ranking, except that the predicted value for the 13th sample was higher than two positive samples but lower than all other positive samples. This explains the very high AUC of the semi‐supervised model.

**Figure 6:**
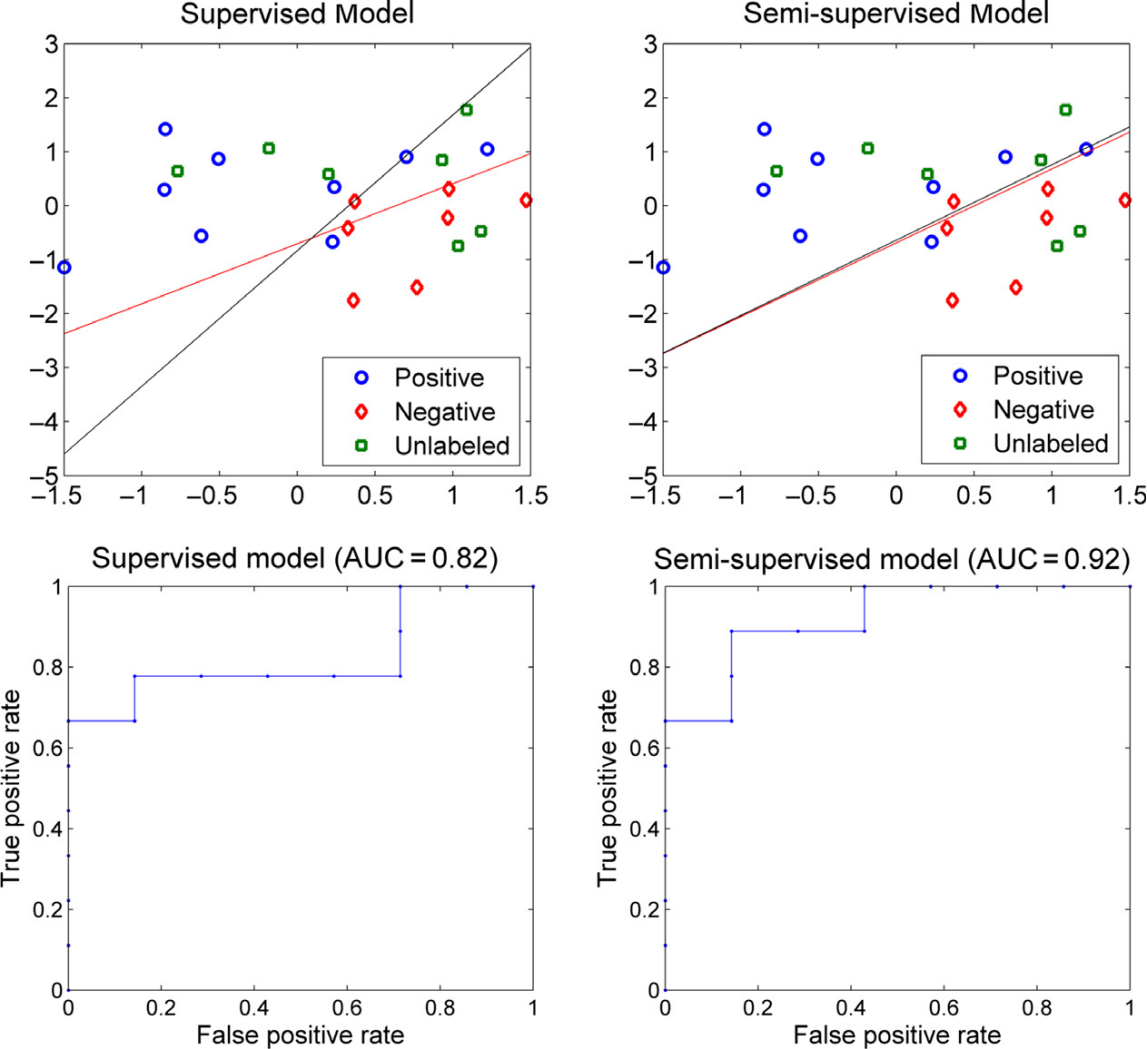
A two‐dimensional example for the comparison between supervised model and semi‐supervised model. The red line represents the hyperplane learnt with all available data, 16 samples for the supervised model and 23 samples for the semi‐supervised model. The two black lines in each panel represent the hyperplanes learnt with sample A or B left out for testing, respectively. Since the two hyperplanes are very close to each other, they appear to be completely overlapped.

We show a two‐dimensional toy example in Figure 6 to make the above explanation more straightforward. In this example, we also had nine positive, seven negative, and seven unlabeled samples. The ratio of positive samples on the unlabeled samples was set to be 0.6. For this example, the supervised model achieved LOOCV AUC of 0.82, whereas the semi‐supervised model achieved an AUC of 0.92. From the hyperplanes learned across different folds of cross‐validation, we noticed that samples A and B were two special samples. They had determined that the area covered by the yellow ellipse belonged to the positive group. If the sample A or B was left out for testing, the supervised model had only one training sample within the yellow ellipse, leaving this area almost uncovered by the training samples. Based on the distribution of training samples, the model learning algorithm was likely to derive a hyperplane classifying the yellow ellipse as negative. Thus, the learned hyperplane deviated considerably from the actual hyperplane represented by the red line in the figure. Thanks to the unlabeled samples, especially the two within the yellow ellipse, the distribution of samples stayed unchanged after leaving sample A or B out for testing. Accordingly, the semi‐supervised model learning algorithm returned a hyperplane very close the actual hyperplane. In addition, we noticed that five of seven unlabeled samples were classified to the positive group in the semi‐supervised model, although the user specified ratio was 4 positive versus 3 negative. This observation implied that the semi‐supervised learning does not force the model to follow the prespecified ratio. On the contrary, it considers the sample distribution in general and finds out the hyperplane minimizing the objective function, even at the cost of some training errors from the unlabeled samples.

Based on the evidence above, we concluded that the semi‐supervised model is more stable and accurate when compared to the supervised model. Due to the high feature‐to‐sample ratio, the distribution of samples in the feature space was not apparent. In such case, the supervised model is sensitive to the elimination of certain samples. For the semi‐supervised model, however, including the unlabeled samples makes the distribution of samples better‐defined. Thus, the semi‐supervised model remains stable even after removing some samples from the training set.

### Blind test on normal hearing infants

Although the semi‐supervised model was indeed more stable across different folds of cross‐validation, one could still argue that the supervised model trained with all of the labeled samples would be as accurate as the semi‐supervised model. To rule out this possibility, we performed a blind test on the NH samples. Theoretically, all of the NH participants had normal hearing and should be categorized to the effective group, although they did not have CELF‐P2 scores to provide a label. As we described in the section Feature extraction, we had constructed a dictionary for the contrast speech versus silence. This dictionary was applied to each NH infant to convert his/her contrast map into a 658‐dimensional feature vector. We then trained a supervised model with the 16 labeled samples, as well as a semi‐supervised model with all of the 23 samples, including seven unlabeled samples, and applied these two classification models to classify the NH infants. The supervised model successfully classified 66.7% of the NH infants as effective, whereas the semi‐supervised model correctly classified 81.0%, which was close to the LOOCV classification accuracy on the labeled samples. This result further confirmed that the semi‐supervised model was more accurate than the supervised model.

### Semi‐supervised model by adding the unlabeled samples one by one

We investigated how the classification performance changed as we added the unlabeled samples one by one. As we changed the set of unlabeled samples to be added to the training, the parameter *r* changed accordingly. If parameter *r* was unknown, we needed to perform nested LOOCV to evaluate the model. For simplicity and consistency, we first trained a semi‐supervised model using all of the 23 samples to assign labels for the unlabeled samples. Subsequently, the predicted labels for the unlabeled samples were used to calculate the parameter *r*, which was used as the input for model training for the experiment in this section. For example, we first added one unlabeled sample, which gave us a sample size of 17 including 16 labeled samples and one unlabeled sample. If this unlabeled sample was an effective CI‐user according to the predictions above, we set parameter *r* to be 1.0. After that, we added the 2nd unlabeled sample. If the 2nd unlabeled sample was an ineffective CI‐user, we set the parameter *r* to be 0.5, given the 1st unlabeled sample was an effective CI‐user. Then, we added the 3rd unlabeled sample, 4th unlabeled sample, etc. Models were tested using the LOOCV approach, since *r* was already determined before the model training. The order in which the samples were added was generated randomly. Considering the possible bias effect of the order, we tried two sets of orders, and added the samples in both forward order and reverse order. The results are shown in Table 5. The sample indexes for the two orders were not the same, for example, the 7th sample from Order 1 and the 7th sample from Order 2 were not the same sample. From Table 5, we found that the classification performance was the same as or very similar to that of the supervised model, when we added only one or two unlabeled samples. Adding the 4 positive unlabeled samples alone also did not improve the classification performance. Meanwhile, the improvement was negligible when only the three negative unlabeled samples were added to the training set. The improvement became significant only when we added both positive and negative unlabeled samples. Furthermore, the performance improved gradually as we added more samples. There seemed to be a jump in performance when we added five or six unlabeled samples. We speculated that adding 5 or 6 unlabeled samples reached a point where the sample distributions were well‐defined and the distinction between the two groups of samples became obvious, which might have explained the performance jump.

**Table 5:** LOOCV performance of the semi‐supervised model when adding the unlabeled samples one by one

Random order	Forward order						Reverse order					
	Sample index	Label	Ratio *r*	AUC	Pcorr	Scorr	Sample index	Label	Ratio *r*	AUC	Pcorr	Scorr
Order 1	1	+	1.0	0.73	0.38	0.44	7	+	1.0	0.73	0.38	0.44
	2	−	0.5	0.73	0.42	0.40	6	+	1.0	0.73	0.38	0.44
	3	+	0.6	0.79	0.50	0.53	5	−	0.6	0.76	0.46	0.44
	4	−	0.5	0.81	0.54	0.56	4	−	0.5	0.81	0.50	0.49
	5	−	0.4	0.90	0.56	0.69	3	+	0.6	0.92	0.56	0.70
	6	+	0.5	0.94	0.57	0.72	2	−	0.5	0.92	0.56	0.70
	7	+	0.6	0.97	0.60	0.76	1	+	0.6	0.97	0.60	0.76
Order 2	1	+	1.0	0.73	0.38	0.44	7	−	0.0	0.73	0.40	0.40
	2	+	1.0	0.73	0.38	0.44	6	−	0.0	0.75	0.43	0.44
	3	+	1.0	0.73	0.38	0.44	5	−	0.0	0.75	0.43	0.44
	4	+	1.0	0.73	0.38	0.44	4	+	0.3	0.78	0.47	0.50
	5	−	0.8	0.87	0.54	0.63	3	+	0.4	0.81	0.50	0.49
	6	−	0.7	0.95	0.59	0.72	2	+	0.5	0.92	0.56	0.70
	7	−	0.6	0.97	0.60	0.76	1	+	0.6	0.97	0.60	0.76

LOOCV, Leave‐One‐Out Cross‐Validation.

### Semi‐supervised model with artificial unlabeled samples

We also investigated whether the improvement in classification performance was dependent on these particular seven unlabeled samples. Could we still obtain a performance improvement if we were given another set of unlabeled samples? We assumed that the labeled samples were good representations of the actual sample populations, for example, CI effective users and CI ineffective users. Under this hypothesis, the mean and standard deviation for each sample population can be estimated using the labeled samples. Thus, the simulated samples were generated by adding white noise to the population mean as formulated in Equation (4).

(4) x=m+alpha∗s∗rand

where *x* is the feature vector for the simulated sample, *m* and *s* are the mean and standard deviation of the labeled samples, respectively, *x, m, s* are 658‐dimensional vectors since there are 658 features for the contrast speech versus silence. *rand* is also a 658‐dimensional vector containing pseudorandom values drawn from the standard normal distribution, which was generated by the *randn* function in MATLAB. *alpha* is a parameter used to control the noise strength. We estimated the mean *m* and standard deviation *s* for the CI‐effective group and CI‐ineffective group separately, and generated five simulated samples for each group. Thus, we had 26 samples, including 16 labeled samples and 10 unlabeled samples. A semi‐supervised model with parameter *r* equal to 0.5 could be trained. Still, the LOOCV approach was used to evaluate the model. For each *alpha*, we performed this experiment 10 times and calculated the mean and standard deviation for the performance measures across the 10 runs. The results are shown in Table 6.

**Table 6:** LOOCV performance of the semi‐supervised model with simulated unlabeled samples

*α*	AUC	Pcorr	Scorr
0.1	0.83 ± 0.02	0.49 ± 0.01	0.53 ± 0.02
0.2	0.82 ± 0.02	0.48 ± 0.02	0.52 ± 0.03
0.3	0.78 ± 0.02	0.45 ± 0.02	0.48 ± 0.03
0.4	0.76 ± 0.01	0.44 ± 0.01	0.45 ± 0.02
0.5	0.76 ± 0.01	0.43 ± 0.03	0.45 ± 0.02

LOOCV, Leave‐One‐Out Cross‐Validation.

We can see that adding the simulated unlabeled samples to the training set helped to improve the classification performance when compared with the supervised model (Table 2). This result suggested that the performance improvement for the semi‐supervised model did not depend on the particular seven unlabeled samples in our original dataset. The semi‐supervised model is expected to outperform the supervised model even with another set of unlabeled samples. Although the semi‐supervised model with the simulated unlabeled samples performed better than the supervised model, it did not perform as good as the semi‐supervised model with the seven actual unlabeled samples (Table 4). This was very reasonable, because the simulated samples were derived from the distribution of the labeled samples, and they did not contribute as much information as the seven actual samples about the actual sample distribution.

### Model with feature selection

**Figure 7:**
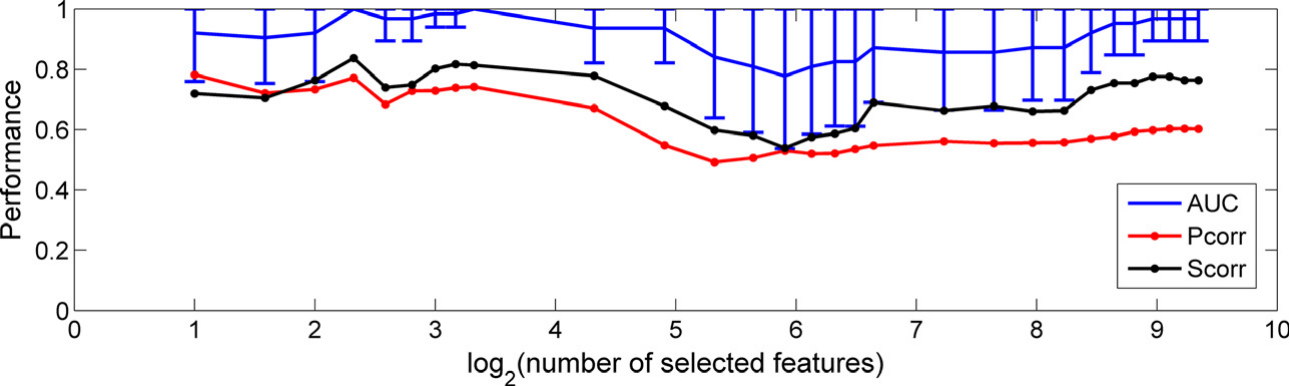
LOOCV performance for the semi‐supervised model using RFE for feature selection with the number of selected features changing from 2 to 650.

In RFE, there are two parameters: the percentage of features to be removed in each iteration and the final number of features to be kept. The first parameter primarily affects the speed of training. If this parameter is small, the training will be slow, and it is less likely to remove relevant features when compared with large values. In our project, we set this parameter at 1%, and required the algorithm to remove one feature at a time if the total number of features was less than 100. For the second parameter, there was not an effective way to determine the threshold before the experiment. Therefore, we tried different thresholds, and performed a LOOCV at each threshold. The classifier performance is shown in Figure 7. We plotted the AUC, Pcorr and Scorr under different thresholds. We also plotted the 95% confidence intervals for the AUCs, which were calculated with the pROC package (Robin et al. 2011). When the number of selected features was set to be 1, the classification problem was not solvable with the default slack variables defined in the SVM^light^ package. Thus, the SVM^light^ package attempted to relax the slack variables gradually. However, the training did not converge after 96 h of computation. From Figure 7, we can see that the confidence intervals of the AUCs overlapped, with the number of selected features ranging from 2 to 650, indicating that the performances, despite random fluctuations, did not change significantly over this interval. In other words, two features would be enough to separate the effective group from the ineffective group. Hence, we set the number of selected features at 2, and performed a LOOCV with RFE for feature selection. We achieved classification accuracy of 93.8%, AUC of 0.92, Pcorr of 0.78 and Scorr of 0.72. This excellent performance confirms that two features are sufficient to separate the effective group from the ineffective group using the semi‐supervised SVM model based on features extracted from the fMRI contrast of speech versus silence.

We also analyzed the performance of the supervised model with RFE for feature selection with the selected number of features set at 2. With the LOOCV approach, we achieved accuracy of 68.8%, AUC of 0.71, Pcorr of 0.56, and Scorr of 0.45, which was much lower than that of the semi‐supervised model.

### Validate the feature selection algorithm

We hypothesized that the two features selected by the RFE algorithm have strong discriminative power in distinguishing effective from ineffective CI users. These selected features may serve as biomarkers for predicting the CI outcomes. Ideally, we would like to predict language performance outcome based on fMRI contrast for speech versus silence. Our features came from the brain regions that responded to the speech stimulus. There might be a chance that all or most of the features were good predictors. Any random selection of two features may yield a good model. If this is true, the features selected by the RFE algorithm will be unreliable and cannot be used as biomarkers. To exclude this possibility, we randomly selected two features from the 658 features to train a semi‐supervised model, and used the LOOCV approach to evaluate the model. We repeated this experiment 100 times and observed a considerable number of runs in which the classification problem was not solvable unless the slack variables were relaxed, indicating that the effective group and ineffective group were not separable based on the two randomly selected features. Furthermore, none of the 100 runs achieved an AUC higher than 0.92, which was the performance when we employed the RFE algorithm for automatic feature selection. Only 3 of the 100 runs achieved an AUC above 0.8. This result indicated that not all features or combinations of features were equally good predictors for the classification of effective versus ineffective CI users. We concluded that the RFE algorithm correctly selected the features with the strongest discriminative power.

### Stability of feature selection

**Figure 8:**
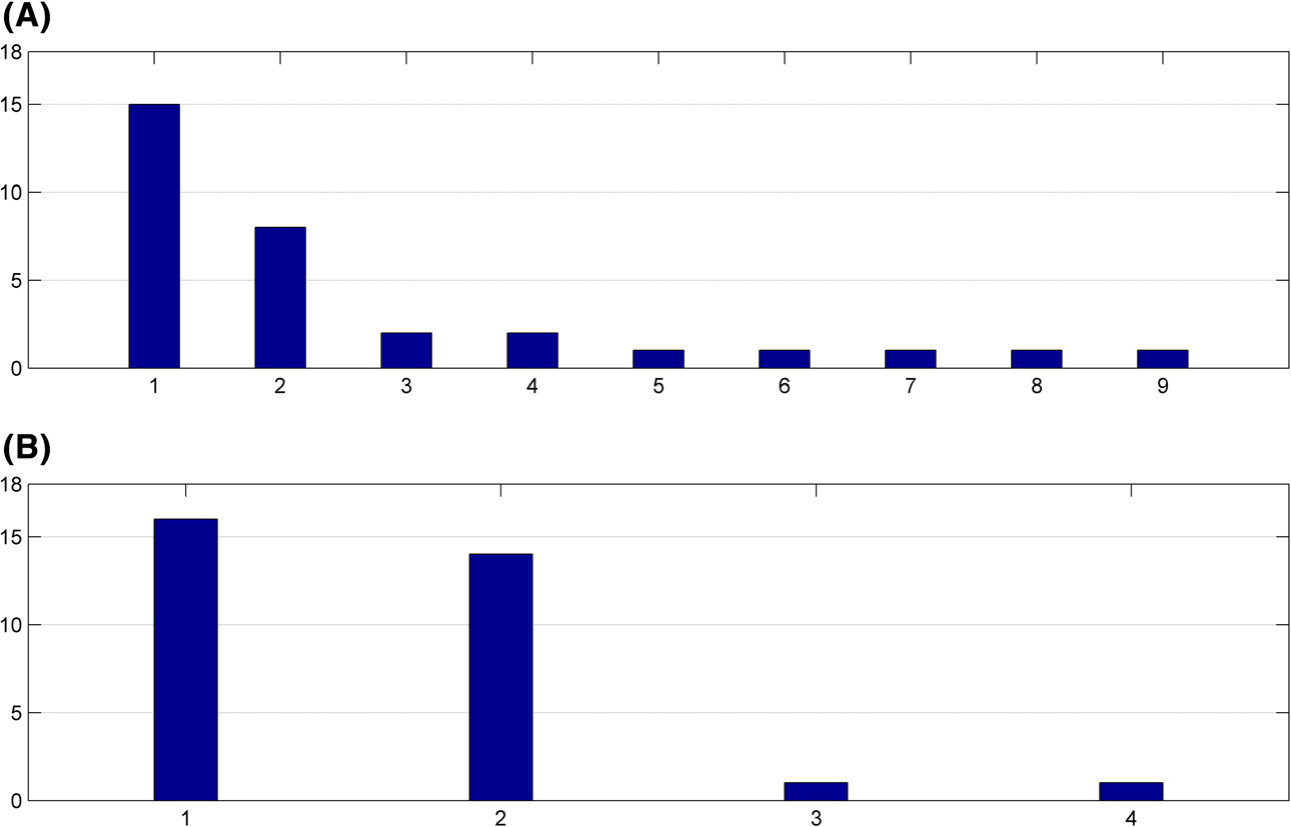
Occurrence frequency of selected features across different folds of cross‐validation. The horizontal axis is the feature index, and the vertical axis is the occurrence frequency of a feature. (A). Supervised model. (B). Semi‐supervised model.

Because the features were selected automatically for each fold of cross‐validation, they might vary across different folds. We had 16 folds of cross‐validation in total, and selected two features at each fold, which led to 32 features in total. We calculated the occurrence frequency for each feature that had occurred for at least once. Besides, there could be multiple features that came from the same brain region and were highly correlated with each other. Such redundancy was attributed to the inherent properties of our feature extraction approach. For example, some characteristic contrast regions might occur in more than one subject as described in the section Feature extraction. In such cases, there were multiple copies of this region in the vocabulary. The features corresponding to such regions were highly correlated. Such features were considered to be redundant and selected interchangeably during the feature selection process. The 64 selected features across different folds of cross‐validation, that is, 32 from the semi‐supervised model and 32 from the supervised model, involved 13 different features. Among the 13 features, a pair of features had a correlation as high as 0.99, and we had verified that this pair of features came from approximately the same brain region. Therefore, we treated this pair of features as one single feature when we calculated the occurrence frequency. Except for this pair of features, the correlations between other pairs of selected features were mostly below 0.8 with a few pairs above 0.8 but below 0.9. Thus, all of the other selected features were treated as different features. As shown in Figure 8, the feature selection was clearly more stable for the semi‐supervised model when compared with the supervised model. Due to the limited number of samples in the supervised learning, the selected features in each fold of cross‐validation might fit the training samples too well to be generalizable to the testing samples. This was a possible explanation for the unstable feature selection and unsatisfactory LOOCV performance for the supervised model.

Comparing Figure 8B with Figure 8A, the most frequently selected feature in Figure 8B and that from Figure 8A were the same feature. However, the second most frequently selected features did not match, and the correlation between these two features was only 0.46. As we mentioned above, features were eliminated one by one at the late stage of RFE. We trained a semi‐supervised model with all the 23 samples, using RFE for feature selection. Let the last 10 features be labeled as A to J. If we reduced the number of selected features from 10 to 2, the first feature to be eliminated was the feature J, followed by feature I and H, etc. The last two features kept in the model were feature A and B. We found that the two most frequently selected features from Figure 8B corresponded to the features A and B, and the second most frequently selected feature in corresponded to the feature D. On the basis of these observations, we concluded that the selected features from the semi‐supervised model were consistent with the selected features from the supervised model. The semi‐supervised model did not select certain features to fit the unlabeled samples. Therefore, we excluded the possibility that some unlabeled samples might be outliers, which forced the feature selection to be fixed on certain features and consequently improved the stability of feature selection. Instead, the improved stability should be attributed to the improved statistical power of the training set due to the inclusion of unlabeled samples.

### Discriminative brain regions

**Figure 9:**
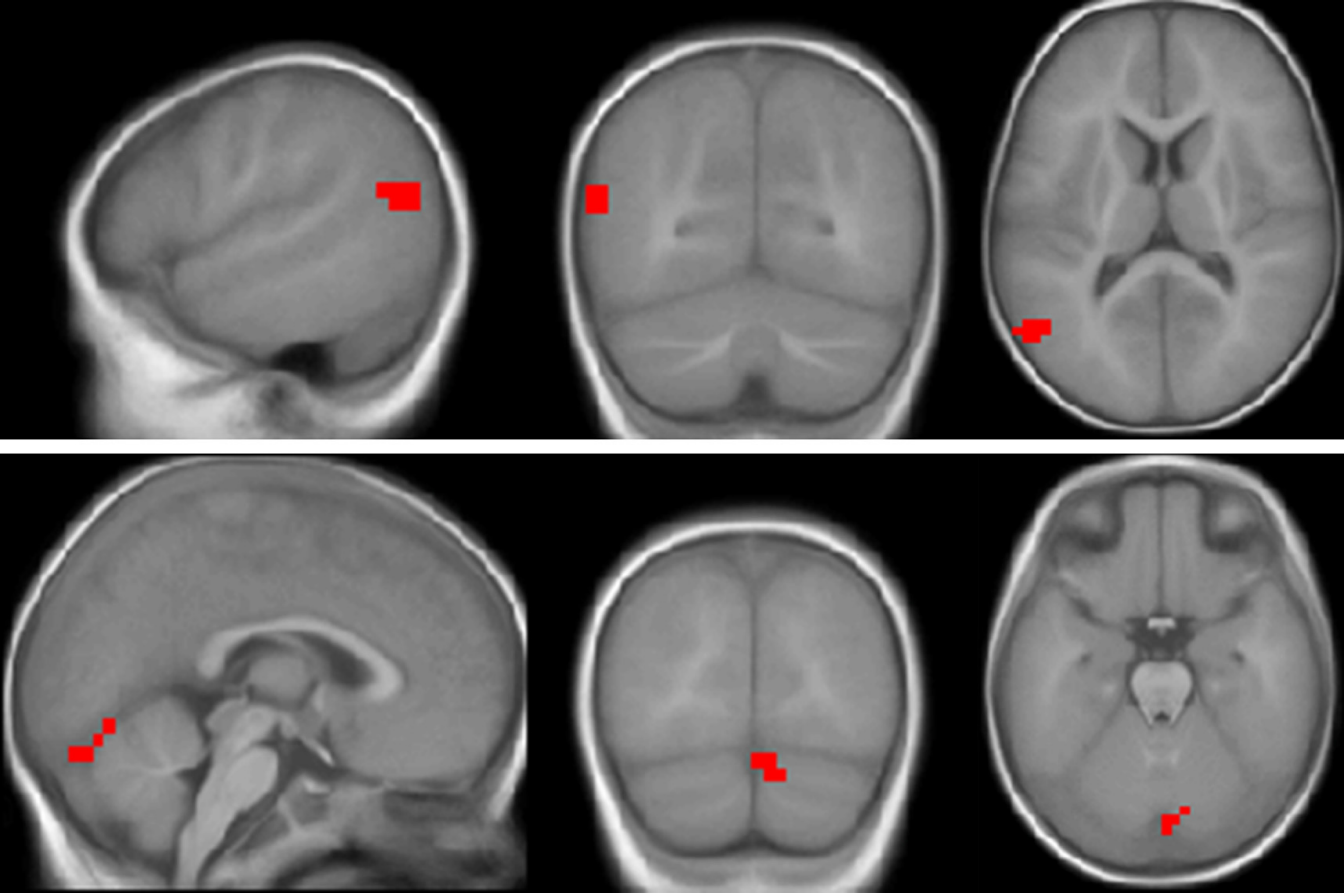
Two discriminative brain regions from the contrast speech versus silence. Images are displayed in neurological orientation using the xjView toolbox (http://www.alivelearn.net/xjview). The coordinate of the center of the first region is (−54, −70, 13). This region is located in an area corresponding to left superior and middle temporal gyri. The second region is located in the right cerebellum. Its central coordinate is (10, −86, −27).

**Figure 10:**
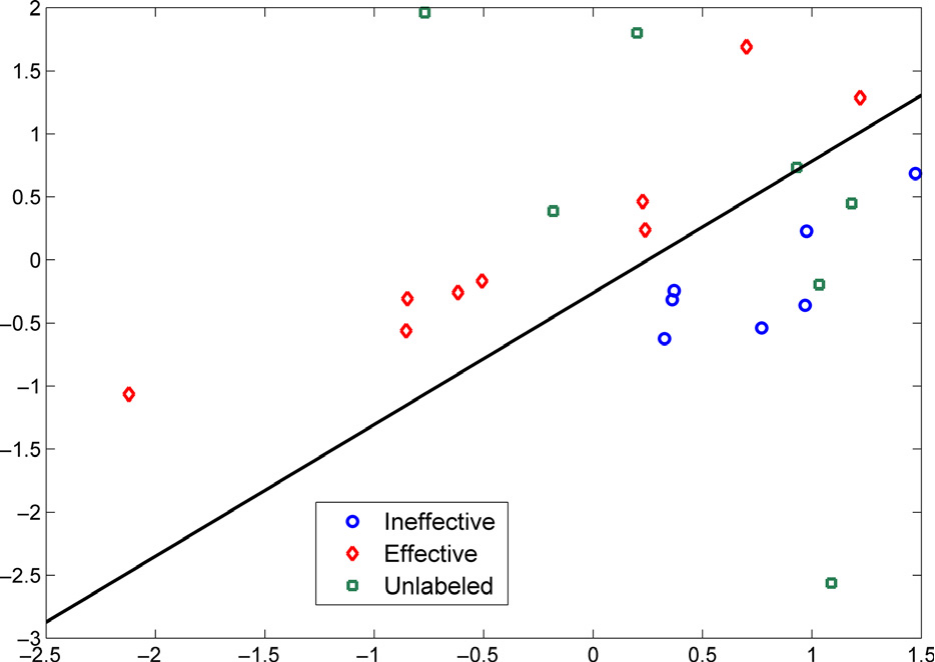
Distribution of samples within the two‐dimensional space. Horizontal axis represents the brain activity in left superior and middle temporal gyri (first brain region in Figure 9), and the vertical axis represents the brain activity in right cerebellum (second brain region in Figure 9).

We trained a semi‐supervised model with all of the 23 subjects and used the RFE for feature selection with the number of selected features set to be 2. Then, we back‐projected the two discriminative brain regions onto the infant template (Altaye et al. 2008) as shown in Figure 9. As we mentioned above, these two features are actually the features 1 and 2 in Figure 8B. The first predictive feature corresponds to a brain region located in the left superior and middle temporal gyri and aligns with our original hypothesis that brain activity in this area might be predictive of outcomes following cochlear implantation in infants. However, using univariate correlation or regression analysis with age at implantation and pre‐implant hearing threshold as covariates, we have not been able to find persuasive predictive value by looking at a region of interest in this part of the brain alone. This implies that one single‐brain region is not enough for the classification. Only the combination of multiple brain regions makes a good prediction for the language function for infants receiving a CI. To verify this observation, we showed the distribution of samples in Figure 10 using the brain activities of the two discriminative brain regions. As we can see, the CI‐effective and CI‐ineffective users were not separable using either a horizontal line or a vertical line, which confirmed that only one brain region was not enough for the classification. Combining these two brain regions, the two groups of individuals were separable by a diagonal line. The other important region is located in the right cerebellum, whose predictive power was underestimated according to our original hypothesis. However, based on a substantial number of scientific publications supporting the role of cerebellum in supporting language functions (see Discussion section), this discovery is not so surprising as it remains unconventional. This finding demonstrates the advantage of machine learning techniques, which can automatically detect the predictive features and draw our attention to features that are important but beyond our prior knowledge.

## Discussion

In this study, we presented a semi‐supervised SVM model for predicting whether or not a prelingually deaf in infant or toddler receiving a CI will develop effective language skills within two years after the surgery. Such prognostic information could be extremely useful and is currently not available to clinicians by any other means. The average cost of cochlear implantation in the United States is $60,000 including device, surgery, and post‐implantation therapy fees (Battey 2007). A reliable predictive tool can guide preoperative counseling, help to calibrate expectations, influence post‐CI speech and language therapy and prevent subsequent disappointment. Noninvasive neurobiological information about developing auditory and language networks in the brain available via fMRI and accurate interpretation of such data using the approach we have developed may also guide timely intervention with CI, and consequently maximize the benefits of CI. The findings of this study demonstrate the remarkable power of a semi‐supervised machine learning approach to the analysis of group fMRI data. Where simple linear regression models between fMRI statistical parameter maps and hearing outcome measures have failed to provide a method by which fMRI data from individual subjects can be used to make a prognosis about possible speech and language outcomes, two features extracted by a machine learning algorithm appear to be able to provide us with a method of doing so. Even with a limited sample size, we have demonstrated a classification accuracy of 93.8% based on two features from brain activation maps of infants listening to natural language during an fMRI scan. Clearly, the methodology needs further exploration and verification with a larger sample size. As the sample size increases, the classification problem becomes more complicated, and it may require additional features to make an accurate classification. But at this stage machine learning classification of fMRI data appears to offer promise in producing an objective prognosis for speech and language outcomes in individual prelingual CI recipients. For the two brain regions highlighted by our current algorithm, we discuss below their functions, and the probable biological mechanisms underpinning their involvement in the classification of effective and ineffective CI users.

### Left superior and middle temporal gyri

Wernicke first described the role of the superior temporal gyrus (STG) in speech perception (Wernicke 1874). Auditorily presented speech stimuli are known to activate the STG (Mummery et al. 1999; Patel et al. 2007; Vannest et al. 2009) whereas lexical/semantic processing requires inputs from the middle temporal gyrus (MTG) (Vandenberghe et al. 1996; Holland et al. 2007). The left STG has been shown to analyze phoneme and word forms (DeWitt and Rauschecker 2013). Jamison et al. (2006) demonstrated, using an fMRI paradigm, that the right STG is specialized for detecting spectral changes even for nonspeech stimuli, whereas the left STG is more highly attuned to temporal variations typical of auditory speech inputs. Recently, Duffy et al. reviewed the utility of the Frequency Modulated Auditory Evoked Response (FMAER) technique in the diagnosis of childhood language impairment (Duffy et al. 2013). The FMAER is based on the detection of quick frequency modulation (FM) changes in speech and its source can be traced to posterior STG bilaterally. The authors found that the FMAER was absent in children with language impairments, especially those with speech comprehension deficits. Children with hearing impairment are known to have language delays and/or deficits (Ching et al. 2010; Yoshinaga‐Itano 2014). Thus, it is reasonable that our SVM models, both the semi‐supervised model and the supervised model, identified the STG as one of the areas differentiating between effective and noneffective CI users based on the variable language proficiency of children with congenital hearing impairment. (Petersen et al. (2013) using positron emission tomography (PET), found that the left STG was activated in a speech comprehension task in postlingually deaf adults but not in prelingually deaf adults. They attributed this finding to the exposure to language in the postlingual HI group. In this study, it is possible that the left STG was identified in the classification of effective versus noneffective CI users as this brain area in the former group may be better tuned to analyzing incoming speech stimuli. At the same time, it is important to note the differences between the two studies before such direct comparisons can be made; for example, in terms of populations studied (adult CI recipients vs. infant/toddler CI candidates), stimulus presentation (monaural vs. binaural), and subject state during acquisition (awake vs. sedated).

In an early landmark PET study aimed at parcellating the brain areas responsible for different aspects of sentence comprehension, Mazoyer et al. (1993) found that the left STG and MTG were activated in response to stories in the native tongue. By comparing different contrasts, they concluded that “the activations in the left middle temporal gyrus… reflect processing beyond the single‐word level” (p. 469), that is, syntactic processing in addition to phonological and lexical analysis. Using PET, Kang et al. (2004) studied the fluorodeoxyglucose (FDG) uptake in the brains of 87 children with congenital hearing impairment ranging from 1 to 15 years of age. Giraud and Lee re‐analyzed their data and found an age‐dependent increase in metabolism in both the superior and middle temporal gyri on the left side (Giraud and Lee 2007). Following a subset of the same participants as they became CI recipients, the authors found the left prefrontal and parietal areas correlated positively with speech perception scores irrespective of the age at implantation or duration of deafness. The authors suggested that these age‐dependent and independent hypermetabolic changes may indicate a two‐step cortical reorganization process in children with congenital HI. In this study, a similar cortical reorganization may have played a role in the effective group resulting in greater language gains post‐implantation. In another study of neural activation using PET, Giraud et al. (2000) found the STG and MTG to be highly active in CI users in response to unrelated sentences. In addition, the anterior portion of the left MTG was also activated in response to a story‐listening task. This observation indicates that the left STG and MTG continue to play a role in speech processing/language acquisition after cochlear implantation. Our results suggest that pre‐CI activation of left STG in response to a natural speech stimulus is one of two key features in the fMRI results that are predictive of later language outcomes for prelingually deaf children receiving a CI.

### Right cerebellum

For over 200 years, the cerebellum was primarily considered to be the center of motor control (Rolando 1809; Holmes 1939). However, this view has been challenged in the past two decades (Habas 2001). In one of the early neuroimaging studies supporting cerebellum as a sensory center, Gao et al. (1996) demonstrated dentate nuclei to be active in response to cutaneous stimulation. Later, overt and covert speech production abilities were associated with cerebellar involvement in both normal (Riecker et al. 2000; Seger et al. 2000; Frings et al. 2006) and disordered (Eckert et al. 2003) populations. However, studies have also indicated cerebellar recruitment in speech reception tasks. Papathanassiou et al., in a PET study, found right cerebellar activation in normal hearing adults in response to a story listening task – similar to the one used in this study (Papathanassiou et al. 2000). Redcay et al., using event‐related fMRI, observed right cerebellar activation in normal hearing toddlers in response to natural speech as compared to silence, a similar contrast to the speech versus silence contrast used in this study (Redcay et al. 2008). Ackermann et al. have also shown cerebellar involvement in the discrimination of vowel duration and voice onset time – important aspects of speech perception (Ackermann et al. 1997). Strelnikov et al. (2006) studied regional cerebral blood flow (rCBF) changes in response to prosodic cues in normal hearing adults. They found right cerebellar activation when participants listened to sentences with intonation patterns. They concluded that the right cerebellum (in addition to the right dorsolateral prefrontal cortex) plays an important role in extracting syntactic and prosodic information (such as pauses and associated pitch changes) from natural sentences.

Fabbro et al. (2000) observed morpho‐syntactic and speech comprehension deficits in patients with focal lesions involving the right cerebellum and vermis. In this study, the right vermis was identified as one of the regions in the cerebellum to be a biomarker of the effective versus ineffective classification of CI users. The vermis has long been considered as the limbic cerebellum (Anand et al. 1959) in that it has cerebro‐cerebellar projections to the cerebral limbic system and it mediates some emotional responses (Timmann and Daum 2007). It is possible that the vermis is tuned to tapping the emotional content in speech stimuli, even under sedation. Alternatively, the right cerebellum may “reflect some basic low‐level aspect of neural processing that may be relevant to speech but cannot be a consequence of accessing the speech system itself” (p. 1761) (Johnsrude et al. 1997).

In a review of cerebellar functions, Marien et al. (2001) observed that “the cerebellum modulates cognitive functioning of at least those parts of the brain to which it is reciprocally connected” and is involved in “various nonmotor language processes such as lexical retrieval, syntax, and language dynamics” (p. 580). This view was recently corroborated by Murdoch based on additional evidence using neuroimaging studies (Murdoch 2010). In the review, Murdoch emphasized the supportive function of the cerebellum in language tasks as opposed to direct involvement – which, in part, may explain the lack of explicit language dysfunction as a result of direct injury to the cerebellum.

### Cerebro‐cerbellar interaction

In this study, two regions – right cerebellar vermis and left cerebrum (temporo‐parietal) – were identified to successfully discriminate between effective and ineffective CI users. This observation may be explained by the right cerebellar‐left cerebral pairing observed in neuroimaging and electrophysiological studies of language lateralization (Desmond et al. 1998; Gronholm et al. 2005). Papathanassiou et al. (2000) found a coupling of the traditional left cerebral language areas with right cerebellar regions for both speech comprehension and production. Strelnikov et al. (2011) found deactivations in the superior temporal gyrus and the right cerebellum in response to degraded speech in normal hearing listeners. Wong et al. (2008) also identified similar areas but with activation instead of deactivation, likely the effect of differences in intelligibility of speech stimuli used in the two studies. Although noise was not explicitly added to speech stimuli in this study, the effects of acoustic MRI scanner noise interleaved with the story segments certainly present a noisy background to the subject who also has a poorly performing auditory system due to congenital deafness.

In this study, we compared the predictive power of three different feature sets, namely VOX, BoW21, and BoW44. The BoW features exhibited much better performance than the VOX features, which was expected as we analyzed in our previous paper (Tan et al. 2013). The classification performance for BoW21 was better than or at least as good as BoW44. BoW21 feature set only included the regions active in the NH infants, whereas BoW44 included the active regions from both NH controls and SNHL patients. It is likely that regions detected in the 21 NH participants included all the relevant brain regions for classification of effective versus ineffective CI users. Regions from the SNHL patients did not add any information for the classification process and simply introduced noise into the classification due to aberrant and inconsistent activation patterns. Also notice that the age and gender were not perfectly matched between the SNHL group and the NH group. However, we did not think that this age/gender difference would weaken our analysis or jeopardize our conclusion as we were classifying effective versus ineffective CI‐users rather than NH versus SNHL. The average age of NH children was 8 months younger than the average age of SNHL children. Although such an age difference during infancy is likely to significantly affect auditory activation patterns, the feature set (BoW21) extracted based on the activation pattern of the NH children was shown to perform well in the classification of effective versus ineffective CI‐users, even in an older group of infants and toddlers. Admittedly, it would be ideal to use a better age/gender‐matched group of NH children, which may help to further improve the classification accuracy. Since our approach works on our current dataset, which represents the worst situation, it is not unreasonable to expect it will work on an ideal dataset.

Correlations (Pcorr and Scorr) for the contrast speech versus silence were considerably higher than the other two contrasts. The human auditory system is highly attuned to human speech sounds and recent theories suggest that the right and left hemisphere structures are specifically tuned to temporal and spectral features of the speech waveform, respectively (Hickok and Poeppel 2007; Ghitza et al. 2012; Poeppel et al. 2012; Wang et al. 2012). Human speech therefore activates a more extensive auditory network than any other form of auditory stimulation. When speech is contrasted with silence we can expect the maximum difference in brain activity to occur within this auditory language network. Therefore, it is not surprising that we find the greatest correlations between the features from the fMRI contrast for speech versus silence with the CELF‐P2 scores as shown in Table 2 and Table 3. Somewhat more surprising is that the auditory system of an infant with severe to profound SNHL still responds maximally to this type of stimulus during fMRI with auditory stimulation prior to cochlear implantation, while she/he is still deaf. The fact that this contrast produces the best classification performance for our chosen model suggests that infants who have the greatest response to human speech (more specifically the voice of the mother of infants and toddlers themselves) are most likely to develop good language capabilities with cochlear implantation.

In this study, we also compared the supervised model with semi‐supervised model. A straightforward explanation was included to illustrate the superiority of the semi‐supervised model, where the unlabeled samples helped to characterize the distribution of samples in the feature space, and therefore the classifier was able to find a more optimal hyperplane to separate the different groups of samples. Furthermore, adding the unlabeled data to the analysis increased the statistical power of training data due to a larger sample size, which helped to highlight the most discriminative features during feature selection. Although the semi‐supervised model showed a remarkable power in the current project, we are not saying a semi‐supervised model is always better than a supervised model. As shown in the section Semi‐supervised model by adding the unlabeled samples one by one, adding either unlabeled positive samples alone or unlabeled negative samples alone produced a classification performance the same as or very close to that of the supervised model. The superiority of a semi‐supervised model over supervised model depends on how much the unlabeled samples contribute to the characterization of the distribution of different groups of samples. Based on our experience, a semi‐supervised model will perform at least as good as a supervised model, and therefore it is always worthwhile to try a semi‐supervised model when unlabeled data are available. Please note this is only an empirical observation, a mathematical proof is needed to support this conclusion. Besides, we also compared the semi‐supervised SVM model with two other related models, namely transductive SVM (TSVM) model and standard logistic regression model. The TSVM model was trained using SVM^*light*^ package with the parameter *r* set to be 0.56, which was the ratio of positive samples among the labeled samples (9 positive samples vs. 7 negative samples). The logistic regression model was trained using the “glmfit” function in MATLAB. The two models were evaluated using the LOOCV approach as well. Results are summarized in Table 7. We can see that the performance of the TSVM model was worse than that of the semi‐supervised SVM model (Table 3) for most of the feature sets. This was expected for reasons as explained below. In our project, we had 16 labeled samples and seven unlabeled samples. Let the number of positive samples within the seven unlabeled samples be N. During the LOOCV process, the TSVM model added the left‐out sample to the training set as an unlabeled sample. If the left‐out sample was positive, the parameter *r* (which was the ratio of positive samples within the unlabeled samples) would be (*N* + 1)/8. If the left‐out sample was negative, the parameter *r* would be N/8. Thus, the parameter *r* kept changing, and there was not a good way to optimize the parameter *r*. For the semi‐supervised model, however, the parameter *r* was a constant (N/7), and it can be optimized using a nested LOOCV as illustrated in Figure 2. Due to the nonoptimal parameter *r*, the performance of the TSVM model was adversely affected. Furthermore, the standard logistic regression model demonstrated almost random classification for most of the feature sets. This was because the number of samples was much smaller than the number of features for our dataset, in which case the logistic regression model without regularization was not estimable. As a result, the logistic regression model was unreliable and performed poorly for the LOOCV.

**Table 7:** LOOCV performance for the transductive SVM (TSVM) model and logistic regression (LR) model

Model	Feature type	Contrast	Sensitivity (%)	Specificity (%)	Accuracy (%)	AUC	Pcorr	Scorr
TSVM	BoW44	Speech vs. Silence	88.9	28.6	62.5	0.80	0.36	0.68
		Noise vs. Silence	77.8	57.1	68.8	0.66	0.32	0.26
		Speech vs. Noise	33.3	71.4	50.0	0.55	0.03	0.09
		Combine	77.8	71.4	75.0	0.76	0.40	0.36
	BoW21	Speech vs. Silence	77.8	42.9	62.5	0.67	0.37	0.33
		Noise vs. Silence	77.8	57.1	68.8	0.74	0.31	0.27
		Speech vs. Noise	44.4	71.4	56.3	0.52	0.00	−0.18
		Combine	88.9	57.1	75.0	0.66	0.37	−0.02
LR	BoW44	Speech vs. Silence	44.4	71.4	56.3	0.51	0.08	−0.07
		Noise vs. Silence	77.8	42.9	62.5	0.62	0.27	0.17
		Speech vs. Noise	77.8	42.9	62.5	0.62	0.08	0.09
		Combine	33.3	71.4	50.0	0.56	−0.07	0.10
	BoW21	Speech vs. Silence	22.2	71.4	43.8	0.43	−0.09	−0.08
		Noise vs. Silence	33.3	71.4	50.0	0.51	−0.02	0.03
		Speech vs. Noise	55.6	42.9	50.0	0.54	−0.17	−0.21
		Combine	44.4	57.1	50.0	0.60	−0.07	0.05

LOOCV, Leave‐One‐Out Cross‐Validation; SVM, Support Vector Machine.

In order to develop a prognostic tool that will eventually be useful clinically, several improvements to the model would be needed as future efforts. First, a larger sample size is needed to train an initial regression model instead of a classification model. We will continue to use the semi‐supervised learning for the regression model, considering the difficulties in recruiting participants and there will still be unlabeled samples whose follow‐up scores are not obtainable among the newly recruited infants. An additional benefit of a larger training dataset would be the possibility of constructing a more complete assessment of patient outcome based on additional measures of speech, language and cognitive ability for each participant. In the current analysis we have classified patients as effective and ineffective CI users based exclusively on the CELF‐P2 scores. While this test provides a comprehensive assessment of language fundamentals in preschool‐aged children, it does not include measures of speech ability or general cognitive function. Clearly the effectiveness of CI usage, even during the early developmental stages, should be based on a broader range of cognitive abilities than language alone, even though a high degree of correlation might be expected among such assessments. Availability of comprehensive neurocognitive data for a larger patient population would allow for a more accurate evaluation of the effectiveness of CI usage, which in turn should improve training of the model and result in a more accurate predictive model.

Another potential research direction might involve improving the feature extraction algorithm. For the current BoW algorithm, we defined the thresholds based on the *P*‐values for the T‐statistics. Optimization of these thresholds might maximize the brain regions while preserving the homogeneity of the contrast intensities within a single region. Finally, we focused on fMRI image features alone in this study. In future work, the age at implantation and the pre‐implant hearing threshold should be included as two additional features in a clinically relevant predictive model, since these variables are known to account for much of the variance in CI outcomes.

## Conclusion

In this work, we have confirmed that our BoW approach is more accurate than the conventional approach for feature extraction to enhance performance of a machine learning approach to making predictions about future clinical outcomes based on fMRI data alone. Not surprisingly, fMRI measures of brain activity stimulated by human speech provided contrasts that were most predictive of language outcomes after cochlear implantation. Semi‐supervised learning made the maximal use of the available data, and provided a stable and accurate classification model for predicting the CI outcomes. Capitalizing on the excellent performance of the semi‐supervised model, we have validated the hypothesis that pre‐implant cortical activation patterns revealed by fMRI during infancy correlate with language performance 2 years after cochlear implantation. By using the recursive feature elimination algorithm for feature selection, we discovered that two features from the fMRI contrast map for speech versus silence were sufficient for classifying effective from ineffective CI users based on our current dataset. We highlighted these two features as discriminative brain regions. One of these two regions is located in an area corresponding to left superior and middle temporal gyris. The left STG is implicated in spectral, phonemic, and lexical processing of human speech. The left MTG is involved in syntactic processing. These observations have been made not only in congenitally hearing impaired children but also in young CI recipients. These findings suggest that the left STG and MTG play an important role in speech processing/language acquisition even in congenitally deaf infants and toddlers. In this study, cortical development in these areas may have played a role in the effective group, resulting in greater language gains post‐implantation. The second region is located in the right cerebellum. The involvement of the right cerebellum in the speech versus silence contrast suggests that it may play a role in extracting syntactic and prosodic information from natural speech and points to the supportive function of the cerebellum in linguistic tasks.

On the basis of this preliminary result, we are optimistic that a reliable machine learning model based on a larger training set can eventually be applied in the clinical setting to provide specific prognostic information to patients considering cochlear implantation.

## Conflict of Interest

None declared.

## Supporting information


**Figure S1.** Timing diagram for fMRI paradigm (This figure is adapted from (Tan et al. 2013) Figure 1).

**Figure S2.** The effect of parameter C on the classification performance for the supervised models.

**Figure S3.** Automatically selected parameter r across different folds of cross‐validation.

**Figure S4.** The effect of parameter C on the classification performance for the semi‐supervised models.
