# Supplementary figures and images for: A semi‐supervised Support Vector Machine model for predicting the language outcomes following cochlear implantation based on pre‐implant brain fMRI imaging
Source: Brain Behav. 2015 Oct 12;5(12):e00391. doi: 10.1002/brb3.391 (PMC4714644; doi:10.1002/brb3.391)

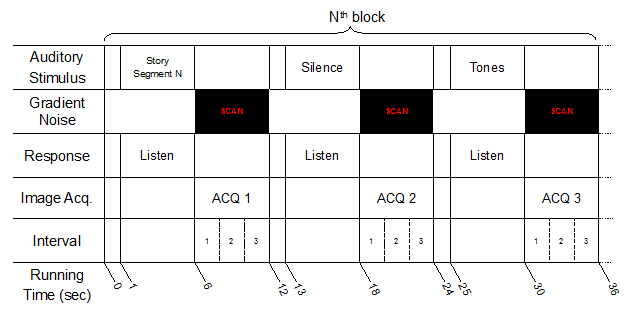

Supplement: Supplementary file 1 — Figure S1. Timing diagram for fMRI paradigm (This figure is adapted from (Tan et al. 2013) Figure 1). [file BRB3-5-e00391-s001.jpg]

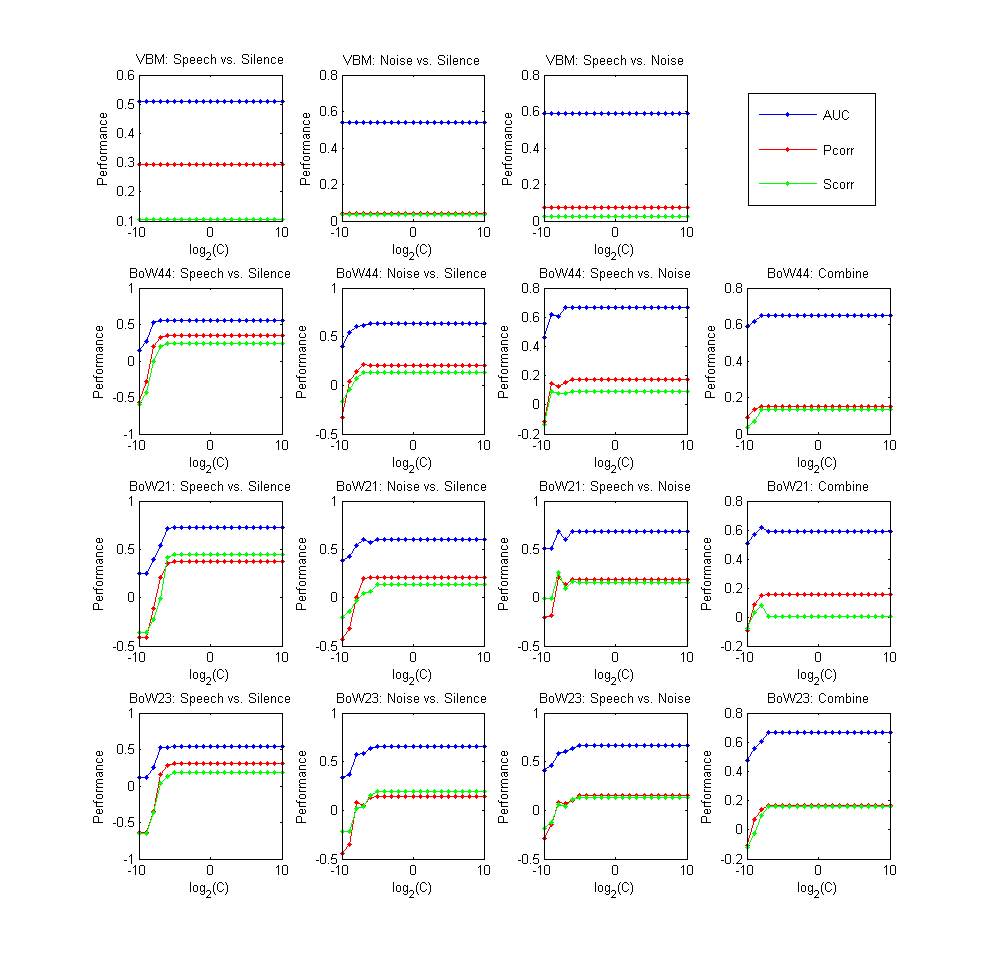

Supplement: Supplementary file 2 — Figure S2. The effect of parameter C on the classification performance for the supervised models. [file BRB3-5-e00391-s002.tif]

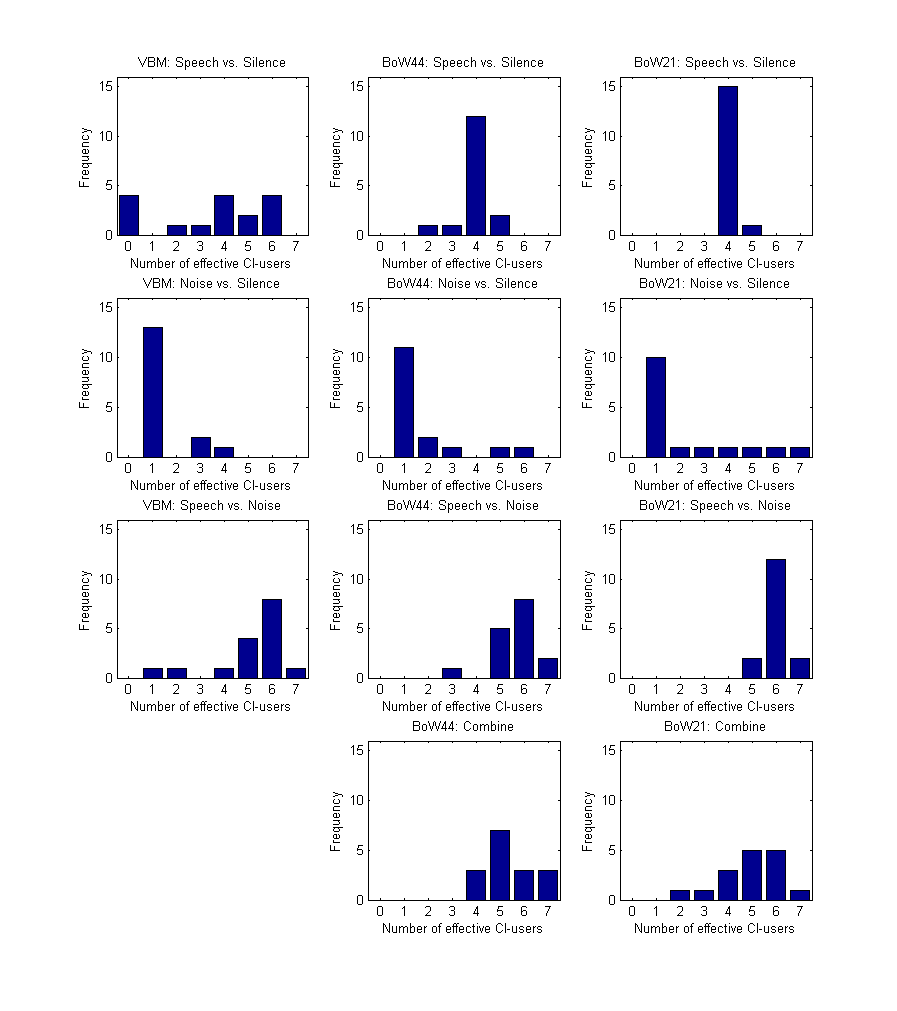

Supplement: Supplementary file 3 — Figure S3. Automatically selected parameter r across different folds of cross‐validation. [file BRB3-5-e00391-s003.tif]

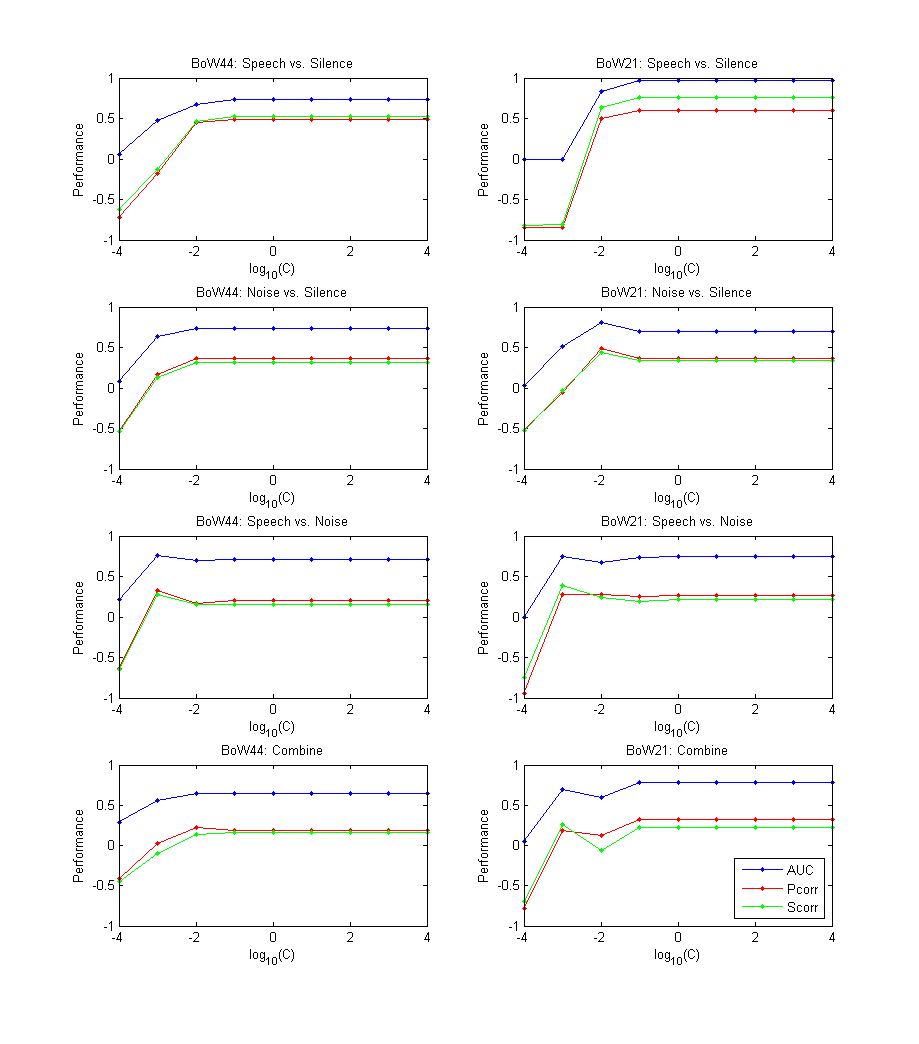

Supplement: Supplementary file 4 — Figure S4. The effect of parameter C on the classification performance for the semi‐supervised models. [file BRB3-5-e00391-s004.tif]
